# Isoform-specific hyperactivation of calpain-2 occurs presymptomatically at the synapse in Alzheimer’s disease mice and correlates with memory deficits in human subjects

**DOI:** 10.1038/s41598-018-31073-6

**Published:** 2018-09-03

**Authors:** Faraz Ahmad, Debajyoti Das, Reddy Peera Kommaddi, Latha Diwakar, Ruturaj Gowaikar, Khader Valli Rupanagudi, David A. Bennett, Vijayalakshmi Ravindranath

**Affiliations:** 1 Centre for Neuroscience, Indian Institute of Science, Bangalore, 560012 India; 2 Rush Alzheimer’s Disease Center, Rush University Medical Center, Chicago, IL 60612 USA; 3 Centre for Brain Research, Indian Institute of Science, Bangalore, 560012 India

## Abstract

Calpain hyperactivation is implicated in late-stages of neurodegenerative diseases including Alzheimer’s disease (AD). However, calpains are also critical for synaptic function and plasticity, and hence memory formation and learning. Since synaptic deficits appear early in AD pathogenesis prior to appearance of overt disease symptoms, we examined if localized dysregulation of calpain-1 and/or 2 contributes to early synaptic dysfunction in AD. Increased activity of synaptosomal calpain-2, but not calpain-1 was observed in presymptomatic 1 month old APP^swe^/PS1ΔE9 mice (a mouse model of AD) which have no evident pathological or behavioural hallmarks of AD and persisted up to 10 months of age. However, total cellular levels of calpain-2 remained unaffected. Moreover, synaptosomal calpain-2 was hyperactivated in frontal neocortical tissue samples of post-mortem brains of AD-dementia subjects and correlated significantly with decline in tests for cognitive and memory functions, and increase in levels of β-amyloid deposits in brain. We conclude that isoform-specific hyperactivation of calpain-2, but not calpain-1 occurs at the synapse early in the pathogenesis of AD potentially contributing to the deregulation of synaptic signaling in AD. Our findings would be important in paving the way for potential therapeutic strategies for amelioration of cognitive deficits observed in ageing-related dementia disorders like AD.

## Introduction

Alzheimer’s disease is one of the major causes of dementia in elderly across the world with no cures available currently. Recent studies provide ample evidence of synaptic dysfunction as a key feature of the disease. Indeed, synaptic dysfunction is evident in early stages of AD when there is little β-amyloid deposition or neuronal death^1,2^. Multiple mechanisms are thought to be involved in synaptic dysfunction in AD pathogenesis, however the molecular players are largely unknown.

Calpains are calcium dependent, non-lysosomal neutral proteases that catalyze limited proteolysis (generally not more than one or two peptide bonds) of substrate proteins^3^. Calpains 1 and 2 are the major isoforms of calpain expressed in brain and, are ubiquitously and uniformly distributed in neurons and glia^4^. Hyperactivity of calpains has been linked to both apoptotic and necrotic neuronal death in several *in vitro* and *in vivo* models of neurodegeneration including AD^5^. Not surprisingly then, several groups have shown global dysregulation of calpain activity in AD model systems and human post-mortem brains^6,7^. Interestingly, calpain is also thought to be involved in both amyloidogenic^8^ and non-amyloidogenic^9^ processing of APP as well as in tau cleavage^10^ and hyperphosphorylation^11^.

Calpains are also involved in normal physiological functions including synaptic functions like organization of neurotransmitter receptors, cytoskeletal dynamics, neurotransmitter release as well as local protein translation^4^. Moreover, recent studies using neuronal cultures exposed to β-amyloid peptides has revealed that calpain may also be linked to deregulation of BDNF signaling through TrkB receptors, potentially leading to synaptic dysfunction^12,13^.

Hence, in spite of evidence for early synaptic deficits in AD and the critical roles played by calpains in synapse physiology it is indeed surprising that synapse-specific deregulation of calpains has not been studied in detail in AD pathology. This is of critical importance since synapse-specific dysregulation of calpains could potentially contribute as an early pathogenic event in AD, and hence can be exploited as a target for potential disease-modifying therapeutic measures. Hence, we examined the status of calpains 1 and 2 in an isoform-specific manner in the synapse during early stage of AD pathogenesis using APP^swe^/PS1ΔE9 mice, a well characterized mouse model of AD^14^ and then used post mortem human brain samples to determine if these observations seen in mouse model of AD could be extrapolated to AD as seen in human subjects.

## Materials and Methods

### Antibodies and reagents

Primary antibodies against calpain-1 and 2 and TrkB were purchased from Cell Signaling Technology, Danvers, MA, USA and anti-β-tubulin antibody was from Sigma, St. Louis, MO, USA. Anti-calpain-1 antibody used for immunoprecipitation of calpain-1 was procured from Millipore. Purified anti-β-Amyloid, 1–16 antibody (6E10) (Cat. No. 803003; RRID: AB_2564652) was obtained from BioLegend Inc., USA. Immunoprecipitation of calpain-1 and calpain-2 was performed using Dynabeads Protein G and Protein A, respectively (Life Technologies, CA, USA) and calpain activity assay was performed using a fluorogenic substrate N-succinyl-Leu-Leu-Val-Tyr-7-amino-4-methylcoumarin (Suc-LLVY-AMC; Farmingdale, NY, USA). Calpain inhibitor, MDL 28170 was procured from Sigma-Aldrich. All other chemicals and reagents used were of analytical grade and obtained from either Sigma-Aldrich or Merck.

### Animals

Transgenic mice (APP^Swe^/PS1ΔE9)85Dbo/J (https://www.jax.org/strain/005864^14^;) expressing chimeric mouse/human amyloid precursor protein with Swedish mutations (K595N-M596L) and mutant human presenilin 1 (exon 9 deletion) served as model system for AD. Animals were genotyped for the presence of transgene as described previously^15^. Three age groups of male APP/PS1 mice, 1–1.5 months (adolescent) and 3–4 months (young adult); both of which show little overt behavioral or pathological phenotype of AD, and 10–12 months old (middle aged), which have overt behavioral deficits and plaque pathology were used. Aged matched male WT littermates were used as controls. All experiments involving animals were performed in accordance with institutional guidelines for the use and care of animals after approval from the Institutional Animal Ethics Committee (IAEC), Central Animal Facility, Indian Institute of Science. Only male mice were used for the study and were housed at ambient temperature of 25 °C under 12 hr light-dark cycle with free access to food chow and drinking water.

### Human post mortem tissues

Frontal neocortical tissue samples from post mortem brains, 12 each of subjects with AD and MCI (mild cognitive impairment) and aged matched control subjects with no cognitive impairment (NCI) were procured from participants in the Religious Orders Study of the Rush Alzheimer’s Disease Centre, Chicago, USA (Supplementary Table 1). All experiments involving human post mortem tissues were performed in accordance with institutional guidelines and after approvals from the Institutional Review Board (IRB), Rush University Medical Center. Informed consent for experimentation from human subjects had been previously obtained. Details of the clinical and neuropathological evaluation have been previously reported^16^.

### Synaptosomal preparation

Synaptosomes were prepared employing a discontinuous sucrose gradient based methodology as described previously^17,18^. This sucrose gradient method has also been employed for preparation of highly enriched and functional pool of synaptosomes from post mortem human brain samples^19,20^. Briefly, tissue from mouse or human brain cortices was dissected out and homogenized in 10 volumes of homogenization buffer (5 mM HEPES buffer, pH 7.4, containing 0.32 M sucrose, 50 mM sodium fluoride, 1 mM sodium orthovanadate, 2 µg/ml aprotinin, 10 µg/ml leupeptin, 7 µg/ml pepstatin A, 100 µg/ml of phenylmethanesulfonyl fluoride (PMSF) and 10 µl/ml of protease inhibitor cocktail) in a Potter-Elvehjem glass tube and pestle. The homogenate was centrifuged at 1000 g at 4 °C for 10 min and the post-nuclear supernatant (PNS) thus obtained was centrifuged again at 12,000 g at 4 °C for 15 min and the pellet was resuspended in 5 mM Tris, pH 8.1 containing 0.32 M sucrose along with protease and phosphatase inhibitors. The re-suspended pellet was then layered over a discontinuous sucrose gradient (0.85–1.0–1.2 M) and centrifuged at 85,000 g for 2 hr at 4 °C. Synaptosomal fraction obtained at the interface of 1 and 1.2 M sucrose was collected, washed twice in 5 mM Tris, pH 8.1 and re-suspended in homogenization buffer without protease inhibitors for further experiments. Synaptosomes, thus prepared were examined using transmission electron microscopy. Synaptosomes were fixed in 3% glutaraldehyde (v/v) and stored at 4 °C for 24 hrs. Sample was subsequently washed with phosphate buffered saline (10 mM, pH 7.4, PBS) and fixed in 1% osmium tetroxide for 90 mins. Further dehydration was done with different grades of alcohol and propylene oxide. The sample was impregnated with 1:1 ratio of propylene oxide: araldite resin, which was increased up to 1:3 ratio followed by pure araldite resin for 3 hrs. Finally, the samples were embedded in flat mould and kept at 60 °C for 48 hr for polymerization. Sections were taken on a Leica ultamicrotome and transferred on to copper grid. Characterization of the ultrastructure of the synaptosomes was performed using the transmission electron microscope (Tecanai, USA).

### Aβ_1-42_-Enzyme-linked immunosorbent assay (ELISA)

Total Aβ_1–42_ level in synaptosomes prepared from the cortex of WT and APP/PS1 mice was measured by ELISA according to the manufacturer instructions (SensoLyte Anti-Mouse/Rat β-Amyloid (1–42) quantitative ELISA Colorimetric, Cat. No. AS-55554, AnaSpec, Inc, Fremont, CA, USA).

### Immunohistochemistry in mouse brain

Mouse brain was fixed in 4% (w/v) buffered paraformaldehyde (PFA). The tissue was processed for paraffin embedding and serial sections (5 μm thick) were cut using microtome (Leica Biosystems Inc., Buffalo Grove, IL, USA). Sections were dewaxed by xylene treatment followed by a brief wash with chloroform to remove the residual xylene. Endogenous peroxidase activity was blocked with methanol containing hydrogen peroxide (3%, v/v) for 30 min. Sections were hydrated in graded alcohol followed by brief washings with water and phosphate buffered saline (10 mM, pH 7.4, PBS). The sections were pressure cooked for 10 min in sodium citrate buffer (pH 6.8). After cooling, the sections were washed with PBS and incubated in normal goat serum for 30 min at room temperature to block non-specific binding. Next, the sections were incubated with antibody against calpain-2 (1:00 dilution) or homer 1 (1:1000 dilution) overnight at 4 °C. After washing with phosphate buffered saline (10 mM, pH 7.4, PBS), the sections were incubated in secondary antibodies conjugated to fluorophore (AlexaFluor647 or AlexaFluor594). Subsequently, the sections were washed and mounted with antifade mounting medium (VECTASHIELD). Image acquisition was performed using 40×/0.75 NA, Zeiss Axio Imager M2 (Carl Zeiss Microscopy, LLC, Thornwood, NY, USA).

### Immunoblotting

Synaptosomal and post-nuclear supernatant (PNS) samples were resolved on SDS-PAGE, electroblotted and immunostained using primary and secondary antibodies. Immunoreactive chemiluminescent signals were detected and the intensities of the bands were quantitated using Bio-Rad Imager. Stripping of blots for re-probing loading control (tubulin) was performed using 62.5 mM Tris, pH 6.8 containing 2% (w/v) SDS and 0.7% (v/v) β-mercaptoethanol for 30 min at 70 °C. Some blots however, were probed for tubulin without stripping.

### Immunoprecipitation

Protein G or A conjugated Dynabeads (Invitrogen) were incubated with anti-calpain-1 and calpain-2 antibodies respectively and synaptosomal samples in calpain assay buffer containing 100 mM HEPES, pH 7.4, 50 mM NaCl, 1% (v/v) Triton X 100, 1 mM sodium fluoride and 1 mM sodium orthovandate overnight at 4 °C. The beads were washed twice with calpain assay buffer at 4 °C and re-suspended in calpain assay buffer and used for isoform-specific calpain assay.

### Isoform-specific activity assay of calpain 1 and 2

Isoform-specific activity of calpain 2 was assayed after immunoprecipitation. Immunopurified calpain-1 or calpain-2 on Protein G or Protein A Dynabeads were re-suspended in calpain assay buffer and activity assay was initiated upon addition of 2 µM or 2 mM CaCl_2_ for calpain-1 or calpain-2, respectively and 50 µM of the fluorogenic substrate N-succinyl-Leu-Leu-Val-Tyr-7-amino-4-methylcoumarin (Suc-LLVY-AMC^21^). Activity of calpain-2 was then followed as an increase in fluorescence (excitation wavelength 360 nm and emission wavelength 460 nm) over time for 30 min at 37 °C. The slope representing the activity of calpain-1 and calpain-2 was calculated from the linear phase of the curve.

### Primary neuronal culture

Brain cortices from post-natal day 1 (P1) APP/PS1 mice and wild-type littermates were used to generate primary neuronal cultures. Mice were genotyped at P0, before sacrificing. Cortical tissue was dissected out using a stereo-microscope (Zeiss Stemi 2000C). Tissue was mechanically dissociated followed by enzymatic digestion with papain. Cells were plated and maintained in Neurobasal-A media (Invitrogen), containing 1X glutamax (Invitrogen), 1X B27 (Invitrogen) and 1 X Penicillin-Streptomycin (Invitrogen).

### Treatment of primary neurons with R-Aβ_42_, AMC peptide substrate, and immunostaining

At 15 days *in-vitro* (DIV) primary neurons were treated with 62.5 nM synthetic Aβ_42_ peptide (R-Aβ_42_ was a generous gift from Dr. Sudipta Maiti, TIFR, Mumbai, India) for 6 hours at 37 °C. Following this, cultures were washed and incubated in fresh media containing 50 µM Suc-LLVY-AMC peptide calpain substrate. Exposure of primary neurons with the calpain substrate was continued for 2 hr at 37 °C. The cells were washed twice with PBS and fixed with 2% paraformaldehyde (PFA) for 15 min at room temperature. Samples were mounted using Prolong Antifade Mounting Medium (Thermo-Fisher Scientific). For immunostaining of calpain-2, homer1, β-amyloid_1–42_ (monoclonal antibody; clone 12F4), cells were permeabilized with 0.3% Triton-X 100 for 5 min and blocked with 3% BSA for 30 min at room temperature. Primary antibodies against calpain-2, homer1 and antibody 12F4 were used at a dilution of 1:100 and incubated overnight at 4 °C. Secondary antibodies (AlexaFluor647, AlexaFluor594, AlexFlour488) were used at a dilution of 1:500 and cells were incubated for 1 hr at room temperature prior to mounting and viewing under the microscope.

### Statistics

Outliers from the dataset were removed using an accepted and widely used statistical method of outlier removal based on median absolute deviation (MAD^22^). Statistical comparisons were made by using either unpaired two tailed Mann-Whitney test or unpaired two tailed t-tests. Multiple groups were compared using one-way analyses of variance followed by post hoc tests with Newman-Keuls correction. Correlation of isoform specific activity of synaptosomal calpain-2 with perceptual orientation and speed, memory and cognitive scores, β-amyloid levels, age at death and post mortem index (pmi) associated with respective subjects was measured using Spearman’s correlation analysis. Results are represented as mean ± standard deviation (SD) and expressed as fold of the respective controls. ‘p’ values < 0.05 were regarded as significant.

### Equipment and Settings

All immunoblot images were acquired on a ChemiDoc^TM^ XRS+ system (Bio-Rad, CA, USA) on chemiluminescent signal accumulation mode and quantitatively analyzed using Image Lab^TM^ software (Bio-Rad, CA, USA). Processing changes to obtain final representative image were done uniformly for all blot images using Microsoft Powerpoint and Photoshop (Adobe).

Fluorescence-based calpain activity assay was recorded in a 96-well plate format on an Infinite R M200 PRO multimode microplate reader (Tecan, Switzerland).

Fluorescent images were captured using a confocal laser-scanning microscope (LSM780, Carl Zeiss) with a 63X oil objective with 1.4 numerical aperture (Plan-Apochromat 63×/1.4 Oil DIC M27, Carl Zeiss). Primary neurons were imaged using a stock confocal microscope (LSM780, Carl Zeiss, Germany), using a series of optical sections along the z-axis (512 × 512-pixel format; image size: 134.95 × 134.95 µm; 3 channels) that were captured with a 63X oil-immersion objective, with no zoom (260 nm pixel size) at 0.4 µm step intervals (z-stack). An imaging speed of 3 and averaging of 2 (with a pixel dwell time of 50.42 µsec) was used. Image bit-depth was 12-bit. A Diode 405-30 laser (405 nm) was used for excitation of Suc-LLVY-AMC peptide (λ_ex_: 380 nm, λ_em_: 460 nm). A 561-nm laser and a 647-nm laser were used to visualize R-Aβ_42_ (λ_ex_: 553 nm, λ_em_: 576 nm) and calpain-2 signal (tagged with AlexaFluor 647 secondary antibody [λ_ex_: 650 nm, λ_em_: 665 nm]), respectively. The stock beam-splitter, MBS 488/561/633 was used. The 561- and 647-nm lasers were used simultaneously (first track, switching lasers every frame), with the emitted signal being detected using photomultiplier tube (PMT). The 405-nm laser was used in the second track, with the signal being detected using gallium arsenide phosphate (GaAsP) detector. All images were acquired under identical conditions, using ZEN 2012 SP4 software (Carl Zeiss). Each z-stack was collapsed into a maximum intensity projection image, prior to intensity analysis. Intensity-based analysis was performed in a blind manner using the Metamorph software (Molecular Devices, CA, USA). For intensity analysis of calpain-2, R-Aβ_42_, and Suc-LLVY-AMC peptide, a mask was created enclosing the entire neuron. The threshold for creating the mask was determined based on average and SD values for every image. Gamma changes and thresholding were introduced using the same software. The resolution for the aforementioned imaging system was approximately 240 nm, which was not enhanced or altered in any manner during subsequent processing. The background intensity values were subtracted to obtain the final values. Images from experimental and control groups were coded for blind analysis.

## Results

### Characterization of synaptosomes and colocalization of calpain-2 with homer1

**Figure 1:**
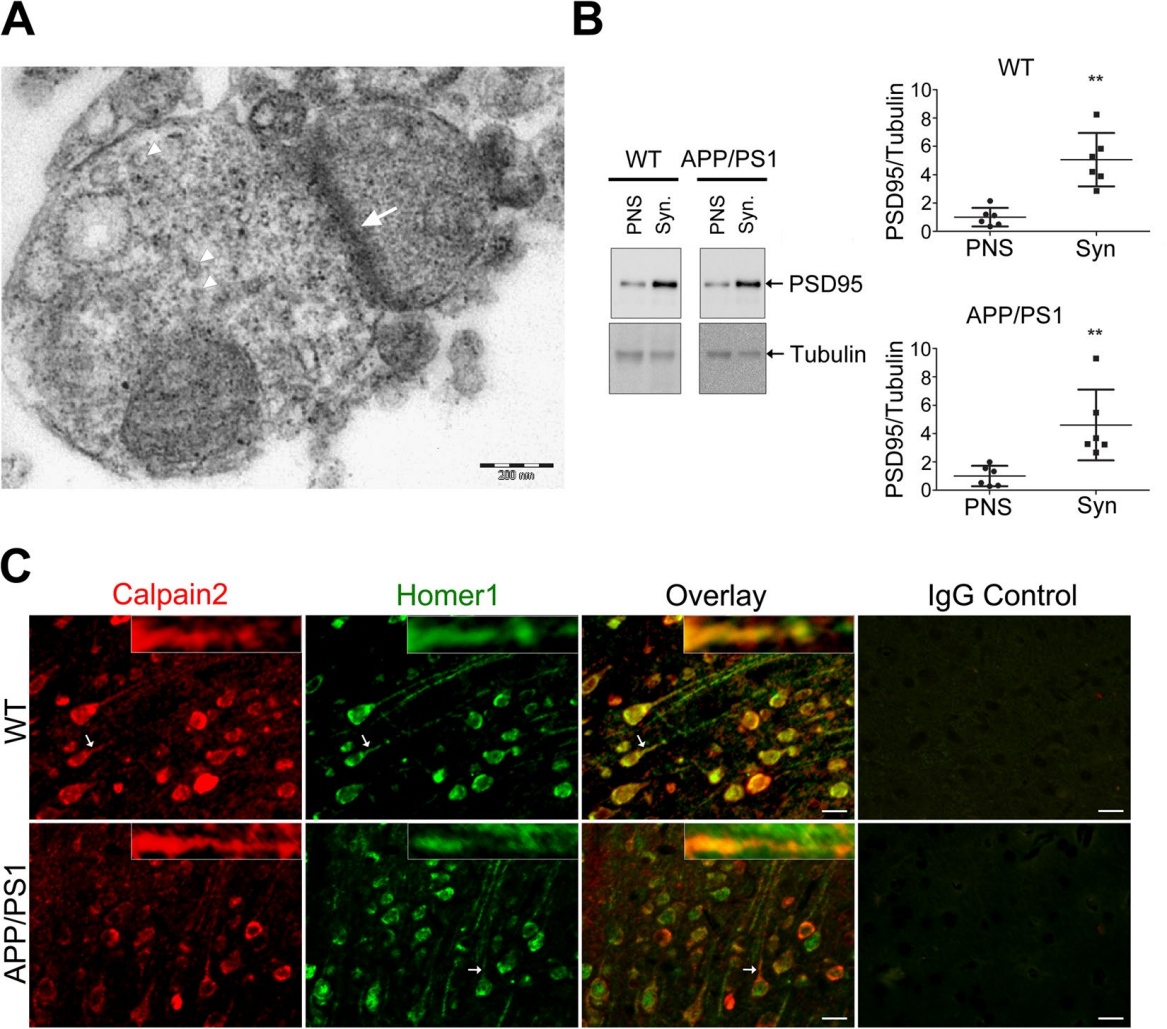
Characterization of synaptosome enrichment and localization of calpain-2 in the synapse. (**A**) Electron micrograph of synaptosome isolated from mouse brain cortex by sucrose density gradient. Synaptic vesicles in the presynapse (arrow head) and the post synaptic density; PSD (arrow) are marked. Scale bar = 200 nm or 0.2 µm (**B**) Enrichment of post synaptic density protein (PSD95) in synaptosomes. The synaptosome isolation was performed as described in Materials and Methods. Post nuclear supernatant (PNS) and synaptosomes (Syn; 8 μg of protein from each fraction/lane) were resolved by SDS-PAGE, and immunoblotted against antibodies to PSD95 and tubulin. Upper panel blots were probed for PSD95. Later, these blots were probed for tubulin (lower panel) without stripping. Densitometric analyses for PSD95 levels were normalized to tubulin. Unpaired two-tailed Mann-Whitney test was used. Results are represented as the mean ± SD (n = 6 mice). (**C**) Colocalization of calpain-2 with homer1 in cortex of WT and APP/PS1 mouse brain. Immunofluorescence staining indicates co-localization of calpain-2 (red) with homer1 (green) in both the cell body and neurites of the neurons. Arrows indicates the immunoreactivity of calpain-2 and homer1 in neurites. Calpain-2 and homer1 co-immunofluorescence was most prominent in neurites as shown in the higher fluorescence micrographs as insets. No staining was seen in mouse cerebral cortex section incubated with IgG control. Scale bar = 75 μm.

Transmission electron micrograph (Fig. 1A) shows the presynaptic terminal with vesicles and the thick layer of postsynaptic density (PSD) indicating that our synaptosomal preparation was intact. PSD95 was enriched 5–7 fold in the synaptosomes as compared to PNS as measured by immunoblotting (Fig. 1B), indicating that the subcellular fractionation enriched the synaptosomal content. We performed this quality control routinely to ensure that the preparation was uniformly good. Colocalization of calpain-2 with homer1 (postsynaptic marker) in mouse brain is depicted in Fig. 1C. A similar co-localization of calpain-2 (w.r.t. homer1) was observed for both the WT and APP/PS1 brains, suggesting appreciable presence of calpain-2 in the neurites and synapses in both WT and APP/PS1 brains.

### Presence of Aβ42 at the synapse

**Figure 2:**
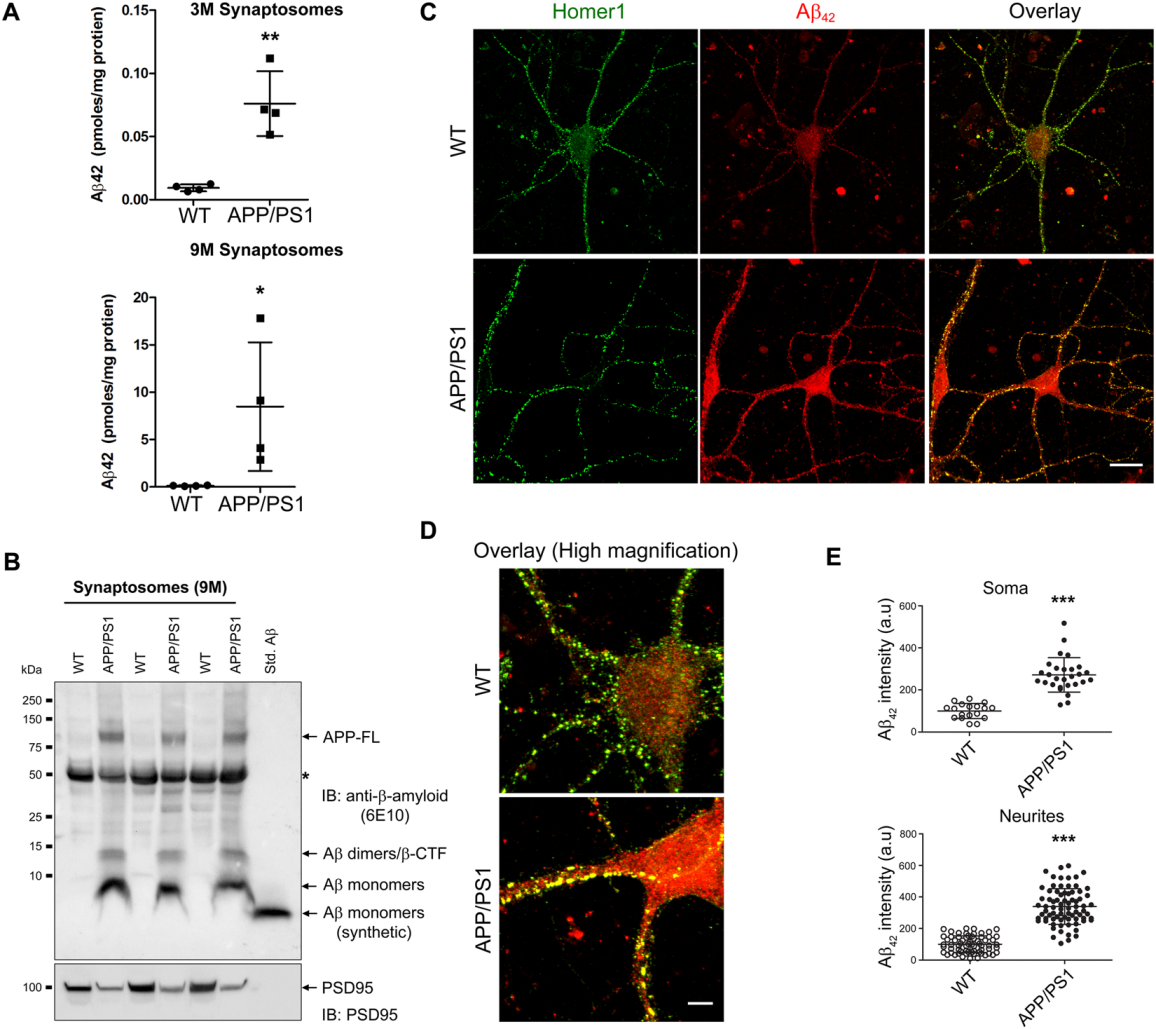
Aβ_1–42_ in cortical synaptosomes and in neurons derived from APP/PS1 mice. (**A**) Total Aβ_1–42_ levels in synaptosomes prepared from WT and APP/PS1 mice at 3 and 9 months of age were measured using ELISA. Data is represented as mean ± SD (n = 4 mice) and was analyzed using two-tailed Mann Whitney t-test. (**B**) The products formed following proteolysis of APP in the synaptosomes were detected by immunoblotting with 6E10 antibody and are represented as Aβ monomers, Aβ dimers/β-CTF and full length APP. Synthetic Aβ_1–42_ was used as standard to confirm the identity of the lower molecular weight bands. ‘*’ indicates a non-specific protein band detected in all samples. (**C**) Representative confocal images of primary neurons from WT and APP/PS1 mice, immunostained against homer1 (green) and Aβ_42_ (red) indicates co-localization of Aβ_42_ with homer1. Scale bar is 20 μm. (**D**) Representative overlay of high magnification confocal images of primary neurons from WT and APP/PS1 mice - Homer1 (green) and Aβ_42_ (red). Scale bar is 5 μm. (**E**) Quantification of Aβ_42_ signal from soma and neurites of both groups is shown. Data is represented as mean ± SD (n = 6–9 neurons and n = 16–36 neurites from each independent experiment). Mann Whitney two-tailed test was used for statistical analysis.

Aβ42 levels were measured in the synaptosomes prepared from cortices of 3 and 9 months old WT and APP/PS1 mice. At 3 months of age synaptosomes from APP/PS1 mice had 0.07 picomoles per milligram protein of Aβ42, while 9 month old animals had 7–8 picomoles per milligram protein. Earlier studies from our lab have reported Aβ42 levels to be 23–26 picomoles per milligram protein in brain lysates at this age^15^, implying presence of around 25–30% of the total Aβ42 in the synaptosomes. It should be mentioned that since the synaptosome preparation was washed extensively it is unlikely that extracellular Aβ42 is present in the synaptosomes. However, at 3 months of age Aβ42 levels in the brain lysate is estimated to be 5 picomoles per milligram protein^17^, thereby representing 1.4% of the total Aβ42 in APP/PS1 mice. Evidence for the presence of Aβ in the synapse was also confirmed by the presence of Aβ in the synaptosomes detected by immunoblotting with 6E10 antibody, which detects APP and its proteolyzed products (Fig. 2B). The immunoblot of synaptosomes from APP/PS1 mice show the presence of full-length APP at ~100 kDa, Aβ peptides (monomer/dimer/ β-CTF). Some non-specific bands were detected at ~55 kDa in both WT and APP/PS1 mice with the 6E10 antibody, but not with other monoclonal antibodies, such as β-actin or β-tubulin etc. We also examined if Aβ42 colocalized with homer1 in the post synaptic terminals in primary neurons. Indeed, appreciable co-localization of Aβ42 with the homer1 puncta was observed in primary neurons derived from APP/PS1 mice (Fig. 2C,D).

### Protein levels of calpain-2 but not calpain-1 are elevated pre-symptomatically in APP/PS1 mice in a synaptosome-specific manner

**Figure 3:**
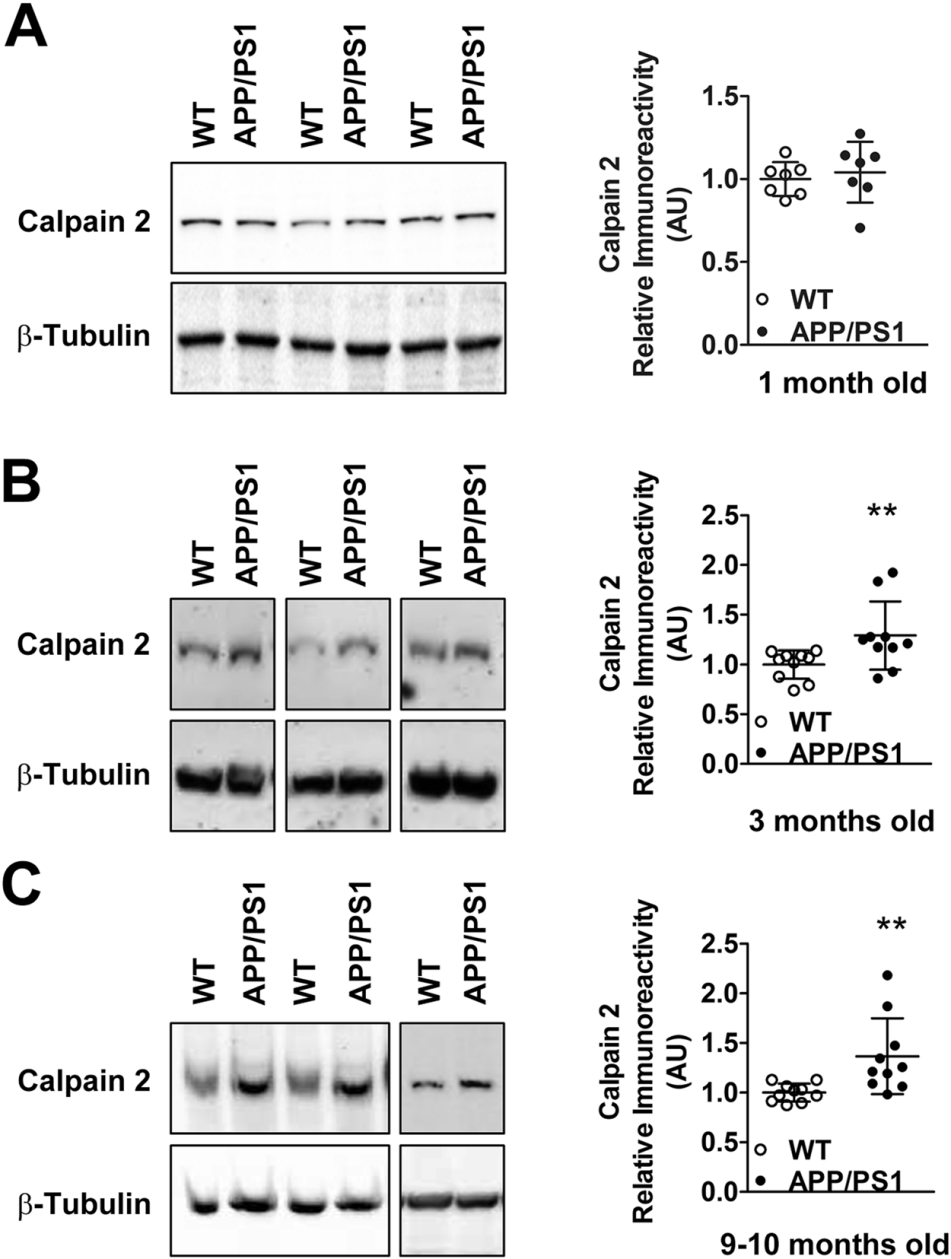
Protein levels of calpain-2 are increased in synaptosomes of APP/PS1 mice early in the disease pathogenesis. While immunoreactive levels of calpain-2 were unaltered in synaptosomes of 1 month old APP/PS1 mice (**A**), calpain-2 levels were significantly increased at 3 months (**B**) and 9–10 months (**C**) of age when compared to age matched WT controls. Values are mean ± SD (n = 7–10 mice) and ** denotes values significantly different from corresponding controls (p < 0.01; Mann Whitney test). In (A), re-probe for tubulin was performed without stripping and in (B), the blot was cut along the dotted line. Full representative blots are also shown in Supplementary data (Supplementary Figs 3–5).

**Figure 4:**
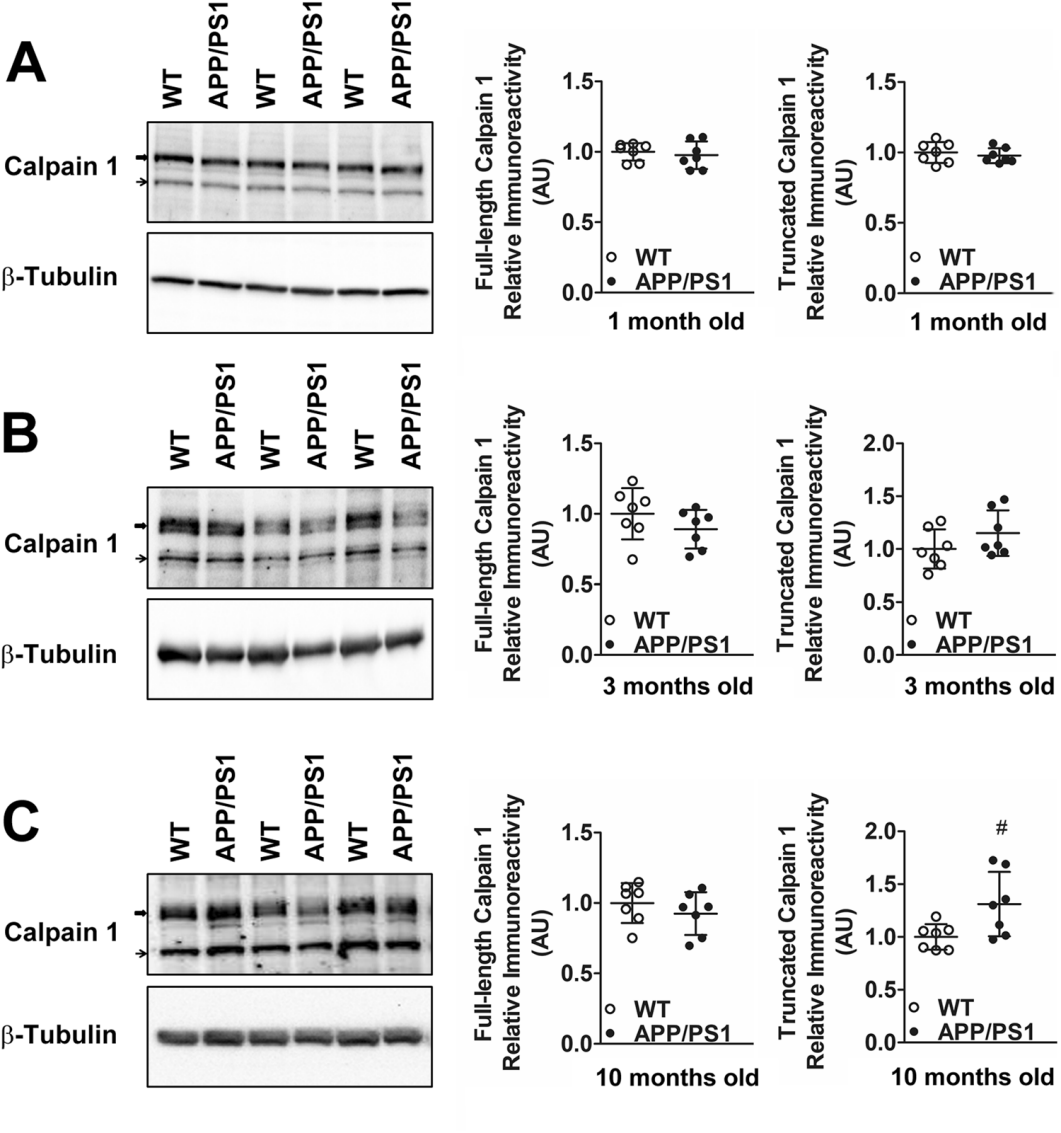
Protein and activity levels of synaptosomal calpain-1 are unaltered in APP/PS1 mice at presymptomatic ages of 1 and 3 months but truncated synaptic calpain-1 levels are elevated at 10 months. Immunoreactive levels of full-length and truncated calpain-1 (marked as block arrows and thin arrows respectively in representative blots) were unaltered in synaptosomes of 1 month (**A**) and 3 months (**B**) old APP/PS1 mice when compared to aged matched WT controls. (**C**) While full-length synaptosomal calpain-1 levels were unaltered at 10 months, truncated calpain-1 levels were significantly increased. Values are mean ± SD (n = 7 mice) and ^‘#’^ denotes p = 0.05 (Mann Whitney test). See full representative blots in Supplementary data (Supplementary Figs 6–8).

Elevated protein levels of calpain-2 in synaptosomal fractions isolated from cortices of APP/PS1 mice were observed from 3 months onward (Figure 3B, n = 10 mice), which continued up to 10–12 months (Fig. 3C, n  = 10 mice). No change was seen at 1 month of age (Fig. 3A). In contrast to calpain-2, which exists as a single form, calpain-1 exists in two forms; a full-length and an autolysed truncated fragment obtained after removal of an N-terminal fragment. The truncated fragment of calpain-1 is regarded as the active form and level of active calpain-1 is generally quantitated as either the truncated form alone or as a ratio of truncated to full-length calpain-1. However, this may be incorrect since it has been shown by X-ray crystallographic structural analysis that the N-terminal peptide of calpain-1 which is truncated does not block the active site in full-length protein^23^, as is the case of many proteases. Removal of the N-terminal sequence from calpain-1 however lowers the calcium requirement for calpain-1 activation^23^. Thus, N-terminal truncation of calpain-1 does not mean conversion of a completely inactive precursor to an active truncated form but rather alteration of full-length calpain-1 with higher calcium requirement for activation to truncated calpain-1 with lower calcium requirement for activation^23,24^. For these reasons, we have quantified both calpain-1 forms (full length and truncated) and represented them individually. Full-length and truncated calpain-1 protein levels were found to be unaltered in synaptosomes isolated from cortices of APP/PS1 mice at 1 and 3 months compared to WT controls (Fig. 4A,B). Interestingly, synaptosomal levels of truncated, but not full-length calpain-1 were increased in APP/PS1 mice of 10 months of age (Fig. 4C), an age when behavioral and pathological symptoms of AD are evident indicating that as the disease progresses there is hyperactivation of both calpain-1 and calpain-2 in the synaptosomes.

**Figure 5:**
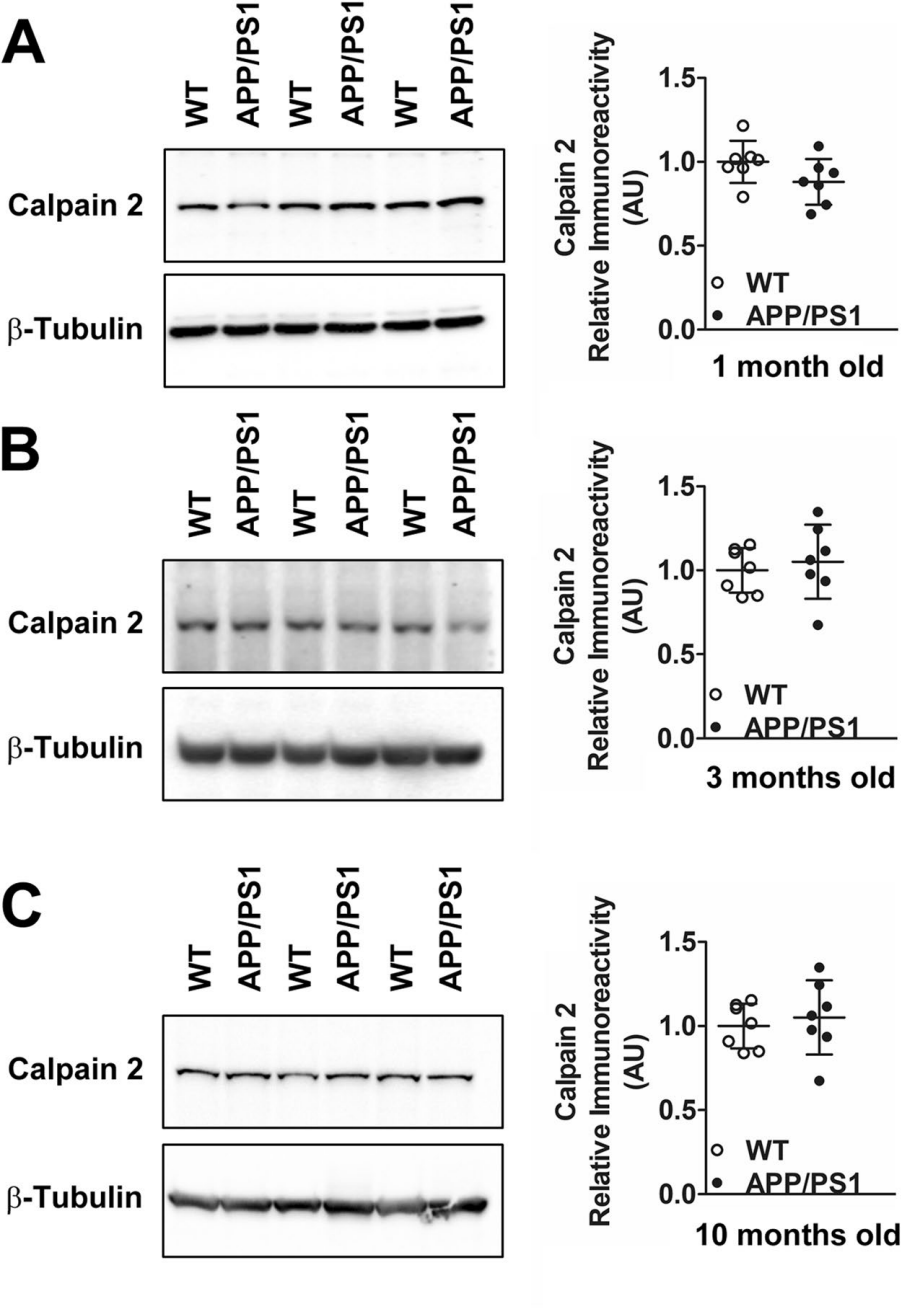
Global protein levels of calpain-2 are unaltered in PNS of APP/PS1 mice at both pre-symptomatic and early symptomatic ages. Global protein levels of calpain-2 as assessed by immunoreactivity in cortical PNS fractions of APP/PS1 mice were similar to aged matched WT controls at ages of 1 month (**A**), 3 months (**B**) and 10 months (**C**). Values are mean ± SD (n = 7 mice) and p values are calculated using Mann Whitney test. See full representative blots in Supplementary data (Supplementary Figs 9–11).

In conclusion, we observe elevated synaptosomal calpain-2 protein levels presymptomatically in APP/PS1 mice from 3 months onwards. However, this presymptomatic early increase in calpain-2 protein levels was isoform specific because a parallel increase in synaptosomal calpain-1 was not observed early in AD progression and appeared only for the truncated form of calpain-1 at 10 months of age when both biochemical and cognitive symptoms are abundantly observed. Moreover, the early increase in calpain-2 was synapse-specific and global calpain-2 levels (as assessed by calpain-2 immunoreactivity in PNS fractions) in cortices of APP/PS1 mice remained unaltered at all ages examined (Fig. 5A–C).

### Synaptosomal calpain-2 is hyperactivated in APP/PS1 mice from an early pre-symptomatic stage in APP/PS1 mice

**Figure 6:**
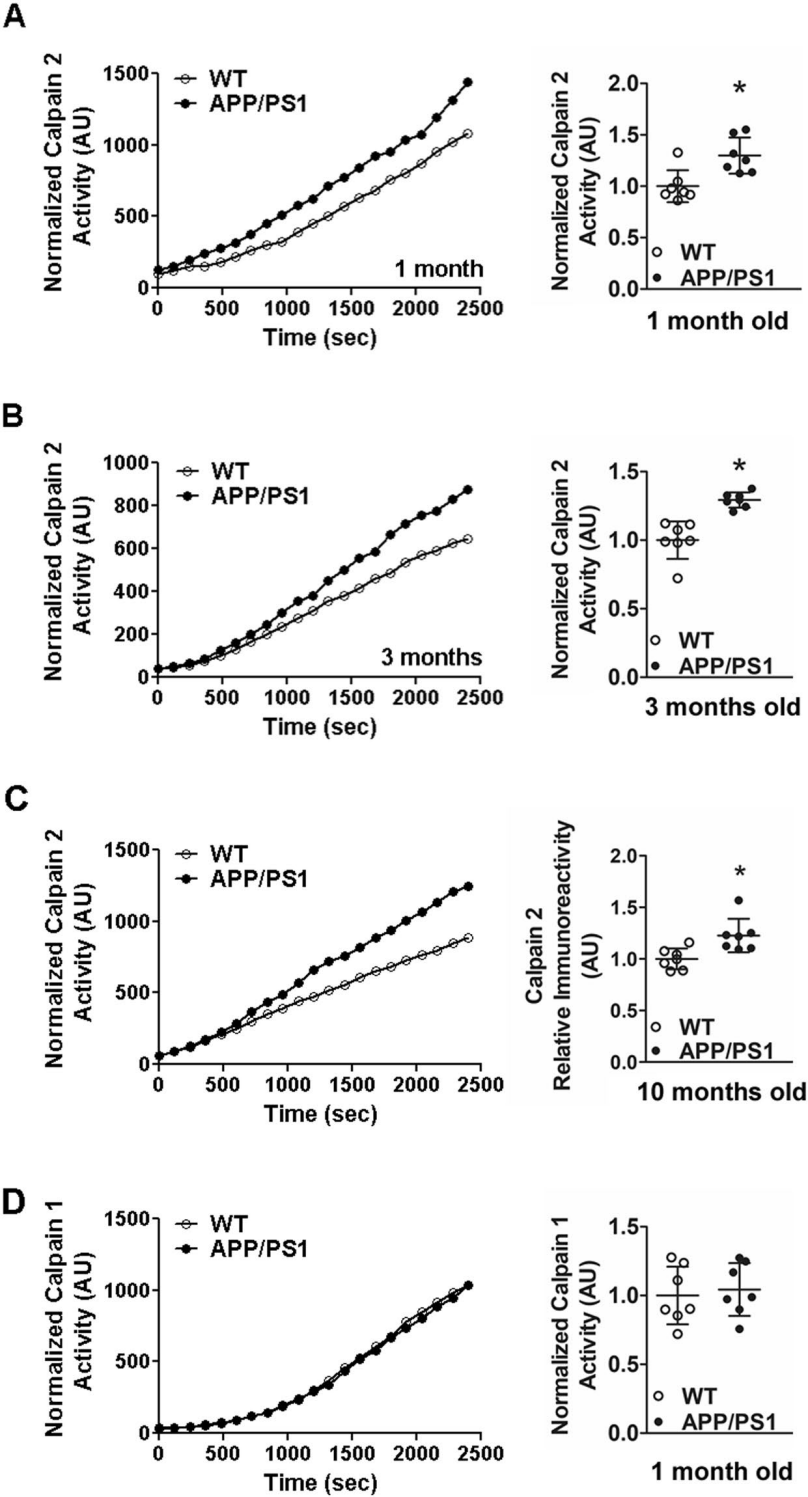
Activity levels of synaptic calpain-2 are upregulated pre-symptomatically in APP/PS1 mice from 1 month onwards. Immunoprecipitation of calpain-2 was employed to assay isoform-specific activity of calpain-2 in synaptosomal samples isolated from brain cortices of APP/PS1 mice. Increased activity of calpain-2 was observed from 1 month of age (**A**), persisting up to 3 months (**B**) and later up to 10 months (**C**). Isoform specific activity of calpain-1 assayed after immunoprecipitation of calpain-1 (See Supplementary Fig. 1) in cortical synaptosomes of APP/PS1 mice was unchanged at 1 month of age (**D**). Each of the individual panels show both the representative activity curves and the cumulative data. Values are mean ± SD (n = 7 mice) and * denotes values significantly different from corresponding controls (p < 0.05; Mann Whitney test). See also Supplementary Fig. 1.

In order to ascertain whether elevated synaptosomal calpain-2 protein levels translate to a concomitant increase in calpain-2 activity, we assayed the isoform-specific activity of synaptosomal calpain-2 following immunoprecipitation with anti-calpain-2 antibody, using a fluorogenic peptide as substrate (Suc-LLVY-AMC^21^; Supplementary Fig. 4). Surprisingly, isoform-specific calpain-2 activity in synaptosomes was found to be elevated from 1 month onwards and persisted at 3 and 10 months of age in APP/PS1 mice (Fig. 6A–C). Although synaptosomal calpain-2 activity increased in 1 month old APP/PS1 mice, we did not detect a concomitant increase in protein level (Fig. 4A). This may be due to contribution of post-translational modification (such as phosphorylation) or association with membrane phospholipids resulting in increase of the activity of calpain-2 (See Discussion).

Because calpain-2 activity but not protein levels are elevated at 1 month of age in synaptosomal fractions isolated from cortices of APP/PS1, we sought to examine whether a similar phenomenon is observed for calpain-1. To this end, isoform-specific activity of synaptosomal calpain-1 from cortices of 1 month old APP/PS1 mice was measured following immunoprecipitation in a manner similar to calpain-2 activity assay (Supplementary Fig. 1). Unlike calpain-2, calpain-1 activity in synaptosomes however was found to be unaltered at 1 month old of age in APP/PS1 mice when compared to wild type controls (Fig. 6D).

In conclusion, our results suggest that the early presymptomatic hyperactivity of synaptosomal calpain-2 in APP/PS1 is isoform specific.

### Levels of full length TrkB, a calpain substrate, are reduced in synaptosomes isolated from APP/PS1 mice

**Figure 7:**
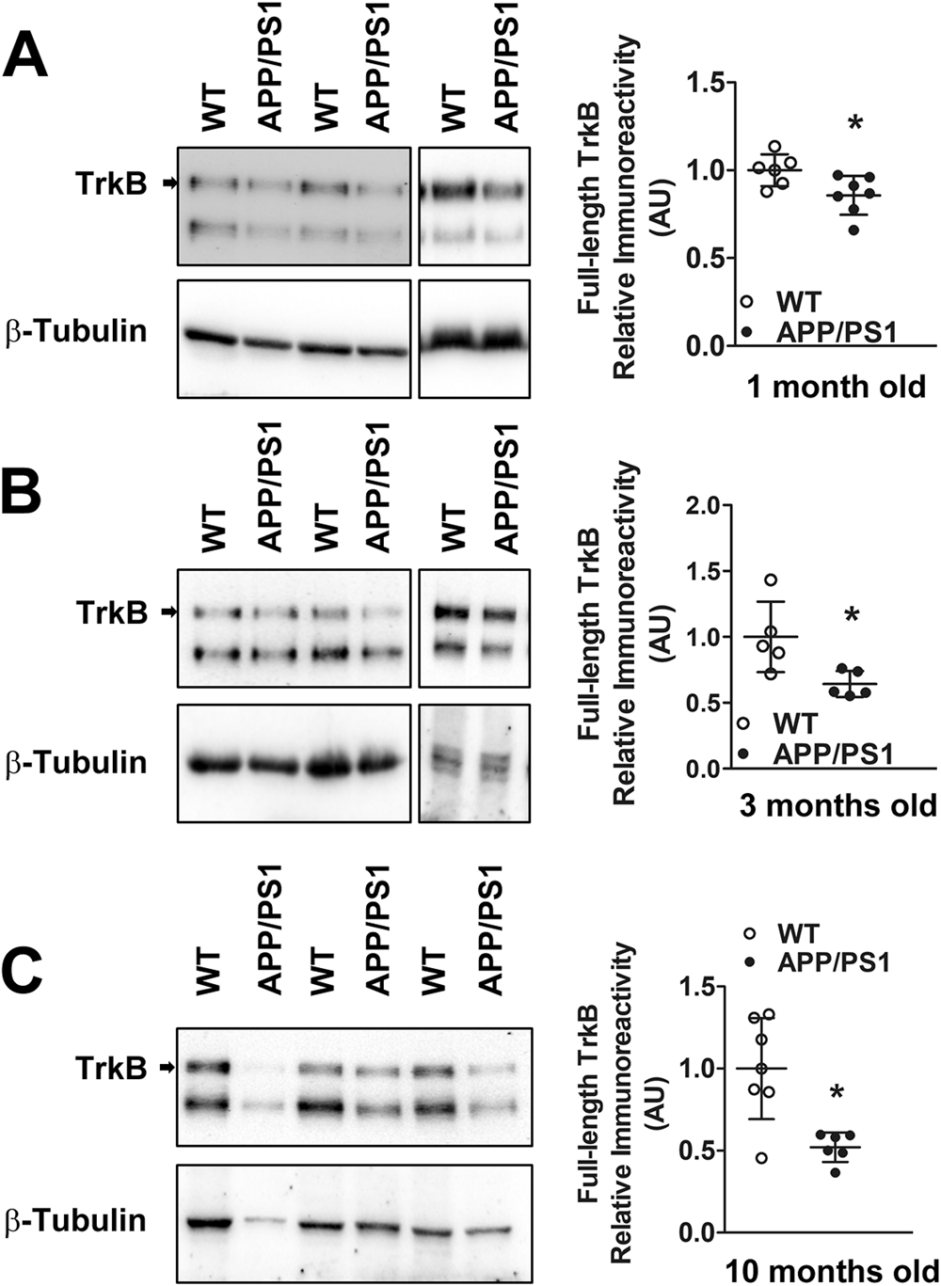
Levels of synaptosomal full-length TrkB, a calpain substrate, is reduced in APP/PS1 mice early in the disease pathogenesis. (**A**) Pre-symptomatic reduction in levels of full-length TrkB, a calpain substrate, was observed in synaptosomes of APP/PS1 mice from 1 month onward. (**B**) Reduced synaptosomal full-length TrkB levels in cortices of APP/PS1 mice persisted up to 10 months of age. Values are mean ± SD (n = 5–7 mice) and * denotes values significantly different from corresponding controls (p < 0.05; Mann Whitney test). See full representative blots in Supplementary data (Supplementary Figs 12,13).

Brain-derived neurotrophic factor (BDNF) plays a critical role in regulating synaptic function and plasticity^25^ and its signaling requires binding to and activating tropomyosin receptor kinase B (TrkB), which is expressed both pre- and post-synaptically^26^. Since TrkB is a known substrate of calpain^12^, we also quantified its level in synaptosomal fraction obtained from cortices of 1 month old APP/PS1 mice. We observed reduced synaptosomal full-length TrkB levels (Fig. 7A), indicating that hyperactivated calpain-2 levels is associated with reduction in full-length TrkB levels at the synapse. Further, there was enhanced loss of synaptosomal TrkB in 10 months old APP/PS1 mice (Fig. 7B), possibly due to contributions from increased levels of both truncated calpain-1 and calpain-2.

### Increase in protein and activity levels of calpain-2 is induced by elevated levels of amyloid beta

**Figure 8:**
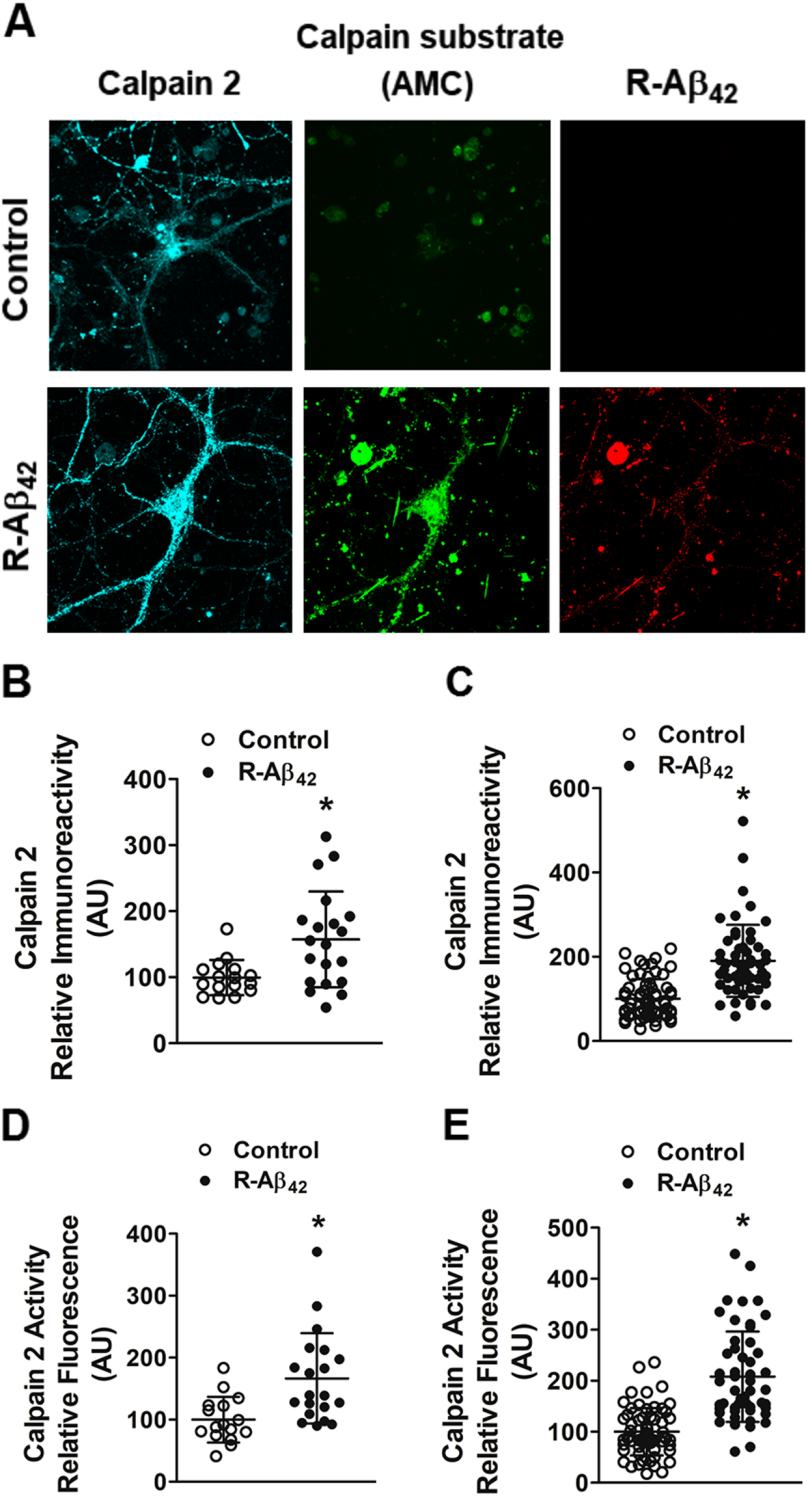
Aβ_42_ enhances both activity and expression of calpain-2. Primary neurons were exposed to R-Aβ_42_ for 6 hours and control neurons were untreated. Primary neurons were then exposed to fluorogenic AMC peptide substrate. (**A**) Representative confocal images are shown, with calpain-2 immunostaining signal in cyan, cleaved AMC peptide signal in green, and R-Aβ_42_ signal in red. Scale bar is 20 µm. (**B**,**C**) Increased expression of calpain-2 levels was observed in both soma and neurites of neurons treated with R-Aβ_42_ compared to control neurons. Increase with calpain-2 levels was associated with a concomitant increase in its activity, as assessed by generation of fluorescent peptide upon cleavage of AMC peptide; both in soma and neurites of primary neurons pre-treated with R-Aβ_42_ (**D**,**E**). Data is represented as mean ± SD (n = 16–18 neurons) and * indicates statistical significance (p < 0.05; Mann Whitney test).

To ascertain that presymptomatic hyperactivity of calpain-2 in APP/PS1 mice is specifically induced by amyloid beta and is not an artefact of overexpression of membrane proteins like APP^27^, we exposed primary cortical neurons to 62.5 nM recombinant Aβ_42_ peptide and observed elevated protein levels of calpain-2 in both neurites and soma and a concomitant increase in its activity as measured by employing the fluorogenic calpain substrate (Fig. 8A–E). This indicates that the increase in calpain-2 protein and activity observed in APP/PS1 mice, the mouse model of AD is specifically caused by elevated amyloid beta deposition and is not an artifact of APP overexpression.

### Increase in calpain-2 protein levels in post-mortem brains of AD patients is synapse-specific

**Figure 9:**
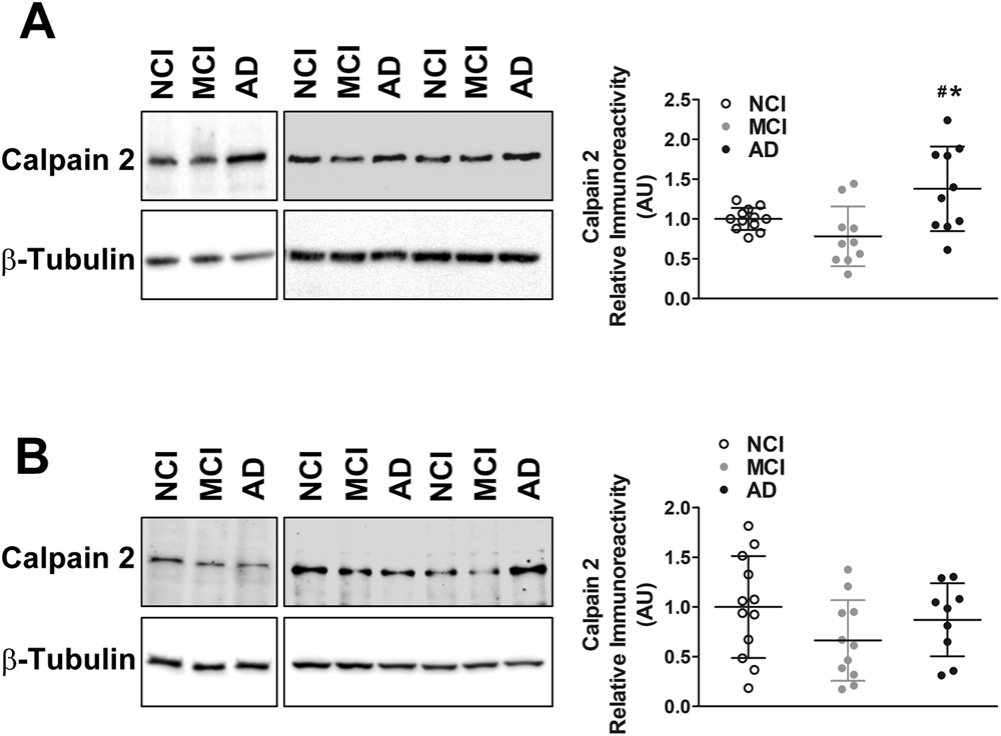
Protein levels of calpain-2 are higher in frontal neocortical tissue samples of post-mortem brains of AD patients in a synaptosome-specific manner. (**A**) Immunoreactive levels of calpain-2 were increased in synaptosomes isolated from post-mortem brains of AD patients when compared to subjects with MCI as well as subjects with NCI. (**B**) Global calpain-2 protein levels were unaltered in post-mortem AD brains. Values are mean ± SD (n = 12 subjects) and * and ^#^ denote significant difference from NCI and MCI groups respectively (p < 0.05; ANOVA followed by post hoc tests with Newman-Keuls correction). See full representative blots in Supplementary data (Supplementary Figs 14,15).

Next, we assessed calpain-2 protein levels in synaptosomes and PNS fractions isolated from frontal neocortical tissue from post-mortem brains of persons with AD dementia compared to subjects with no cognitive impairment (NCI) and mild cognitive impairment (MCI). We observed increased levels of synaptosomal calpain-2 protein in post-mortem AD brains (Fig. 9A), but no change was observed in calpain-2 levels in the PNS (Fig. 9B).

### Synaptosomal calpain-2 is hyperactivated in post-mortem AD brains and activity levels correlate with cognitive and memory parameters and β-amyloid load of the subjects

**Figure 10:**
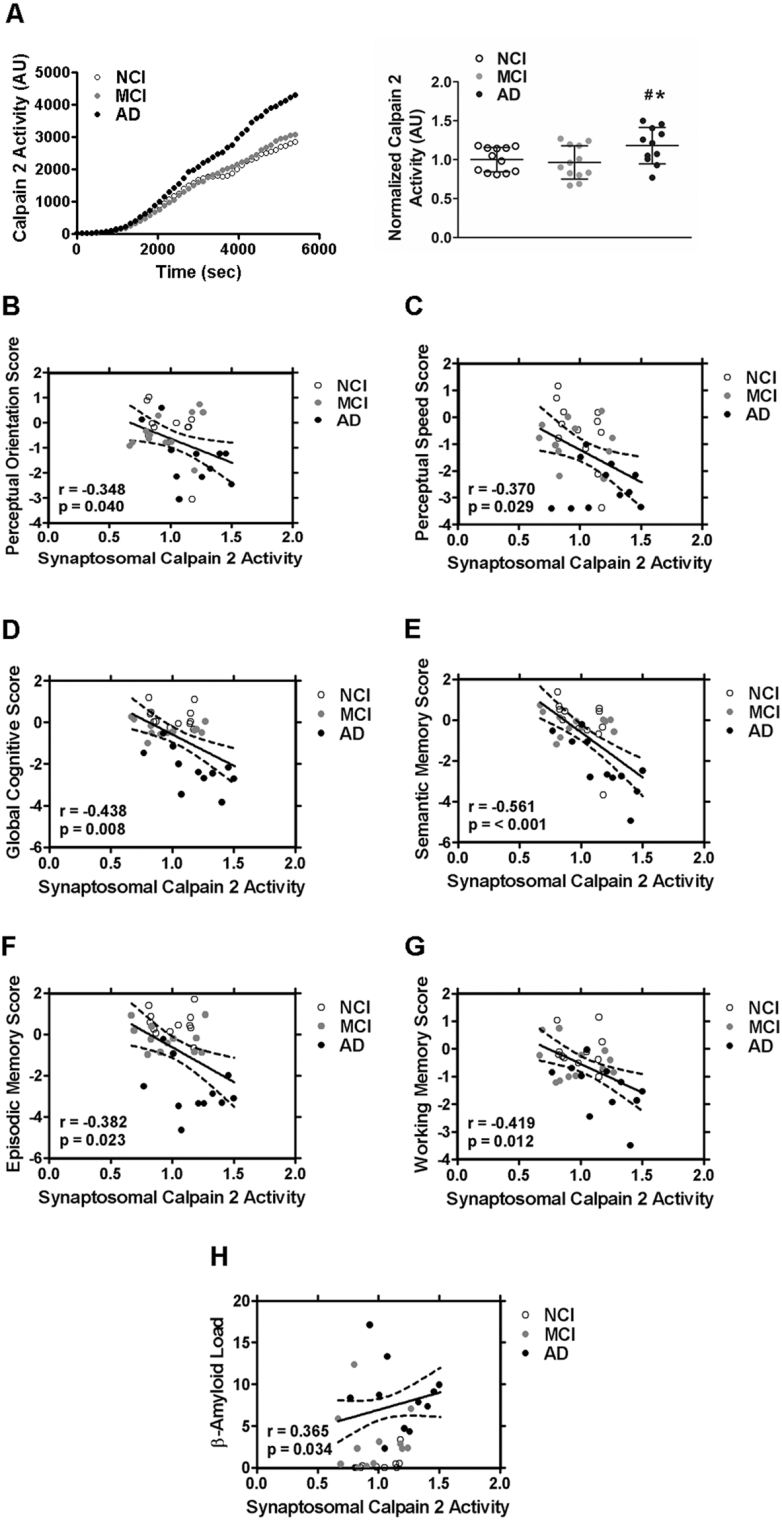
Activity levels of calpain-2 are elevated in synaptosomes isolated from frontal neocortical tissue samples of post-mortem brains from AD patients. (**A**) Activity of synaptosomal calpain-2 immunoprecipitated from post-mortem brains of subjects with AD dementia and was found to be significantly higher compared to subjects with no cognitive impairment (NCI) or mild cognitive impairment (MCI). A significant negative correlation was observed between synaptosomal calpain-2 activity and performance of the subjects in tests for perceptual orientation (**B**) and speed (**C**), global cognition (**D**), semantic memory (**E**), episodic working **(F)** and as well as working memory (**G**) tests. Increase in calpain-2 activity in synaptosomal samples from post-mortem human brains also correlated with increase in amyloid deposits in the brain (**H**). Values are mean ± SD (n = 12 subjects) and * and ^#^ denote significant difference from NCI and MCI groups respectively (p < 0.05; ANOVA followed by post hoc tests with Newman-Keuls correction). See also Supplementary Fig. 2.

Lastly, we assayed isoform-specific calpain-2 activity in synaptosomes isolated from frontal neocortical tissue obtained at post mortem from subjects with AD dementia, controls with NCI and MCI. No correlation was observed between synaptosomal calpain-2 activity and age at death or post mortem interval (Supplementary Fig. 2). We observed higher calpain-2 activity in synaptosomes of AD dementia subjects compared to NCI and MCI (Fig. 10A). Higher synaptosomal calpain-2 activity in AD dementia samples (in comparison to NCI and MCI samples) was associated with reduced global cognition and perceptual speed and visuo-spatial ability; and specifically with decline in performance in tests for episodic, semantic and working memories (Fig. 10B–G). By contrast, higher synaptosomal calpain-2 activity was associated with higher β-amyloid load (Fig. 10E), but not with hyperphosphorylated tau tangles.

## Discussion

We demonstrate using a mouse model of AD (APP^Swe^/PS1ΔE9 mice) that calpain-2 but not calpain-1 is hyperactivated in cortical synaptosomes as early as 1 month of age, much before the onset of the behavioral symptoms and β-amyloid pathology. The increase in calpain-2 activity was reflected in reduced levels of TrkB, a well-characterized substrate of calpain. This increase in calpain-2 levels was synaptosome-specific since we did not observe increase in calpain-2 protein levels in PNS. Synaptosomal calpain-2 hyperactivation was also observed in post mortem brains of human AD dementia subjects and was inversely associated with measures of cognitive function and directly correlated with β-amyloid load.

Several groups have shown higher calpain activity in cellular and animal models of AD, and in human post mortem brains from AD dementia patients^6,7^. In fact, hyperactivation of calpain has been documented to mediate neuronal death and degeneration in a variety of neurodegenerative disorders in later stage of the disease^5^. However, considering both the facts that i) loss of synapse function, structure and morphology is among the earliest events in AD pathogenesis, occurring much before behavioral and pathological symptoms^1,2,28^ and ii) calpain is a critical regulator of synapse function and plasticity^4,29^, we now demonstrate that synaptosomal calpain-2 activity and protein levels increase very early in a synapse-specific manner.

Calpain-2 impacts synaptic function including plasticity through its proteolytic action on substrate synaptic proteins like cytoskeletal elements, channels, receptors and scaffolding proteins signalling^4,30^. Increased activation of calpain-2 in synapses could hence potentially affect a number of critical synaptic functions contributing to AD pathogenesis from an early stage. For example, one critical aspect of synapse function and plasticity that is affected by calpain hyperactivation and contributes to AD pathology would be truncation of TrkB, the central receptor in BDNF signaling pathway^26^ leading to loss of BDNF signalling^12,13^. In fact, we provide evidence that synaptosomal calpain-2 hyperactivation is reflected in reduced levels of synaptosomal TrkB.

We also ascertained that calpain-2 hyperactivation observed in our study is specifically caused by elevated levels of β-amyloid 42 and is not an artifact of APP overexpression as was reported in a recent study^27^. It should also be noted that the study in question provided evidence for hyperactivation of calpain as an artifact of APP overexpression based upon cleavage of p35 to p25. However, we observed increased calpain activity in primary neurons exposed to low concentration of Aβ_42_ indicating clearly the direct effect of Aβ_42_ on calpain activity.

The other interesting observation is the selective increase in calpain-2 but not calpain-1 activity in the prodromal stage of the disease. Although calpains 1 and 2 share several common substrates^31^, recent studies show that they have different functional profiles under physiological^32–34^ and pathological conditions^35–38^. Also, there is evidence for isoform-specific roles for calpains 1 and 2 in induction and consolidation phase of LTP, respectively^34^. The major difference in calpain activation is that under *in vitro* conditions, calpain-1 is activated in presence of micromolar concentrations of calcium while calpain-2 is activated when calcium levels reach millimolar levels^23,29^. Thus, calpain-2 requires order of magnitude higher calcium concentration than calpain-1. However, calpain-2 can be activated when it is localized to the plasma membrane wherein interaction with phospholipids of plasma membrane activate calpain-2 at much lower intracellular calcium concentrations^39^. Calpain-2 can also be phosphorylated by extracellular signal regulated kinase (ERK) resulting in its activation, which occurs independent of calcium concentration^21,40^. Of note, ERK-mediated phosphorylation is calpain-2-specific and does not affect calpain-1 activity^40^. Interestingly, calpain-2, after phosphorylation by ERK, localizes preferentially to dendrites^40^. Further, ERK has been shown to be hyperactivated early in AD pathogenesis^41,42^. Hence, increased activation of ERK in AD could contribute to the synaptosome-specific increase in calpain-2 activity that we observe in APP/PS1 mouse model from 1 month of age. Importantly, this provides an explanation for the increase in activity but not protein levels of calpain-2 in synaptosomes from 1 month old APP/PS1 and also suggests that measurement of calpain activity is a better indicator of calpain status than the relative protein levels.

Our results, using APP/PS1 mice, a widely used mouse model of AD, suggest that at least in the early stage of AD pathogenesis, calpain-2 but not calpain-1 dysregulation is localized to synapses and is not seen in brain lysates. Further, synaptosomal calpain-2 activity increased specifically at prodromal stages of the disease and calpain-1 was largely unaffected in the early stages of AD pathogenesis. Only at 10–12 months of age when overt behavioral and pathological symptoms of AD are evident in these mice, we observed increased levels of truncated but not full length calpain-1. Our study thus contributes to the growing list of evidence demonstrating isoform-specific differences in activation of calpains 1 and 2 in pathophysiological conditions.

Lastly, increased levels of calpain-2 have been observed in post-mortem AD brains in numerous studies^7,43^. Interestingly, increased protein and activity levels of calpain-2 have also been observed in CSF samples of human subjects with AD dementia in late stage of the disease when compared to non-demented controls^44^. We now demonstrate elevated synaptosomal calpain-2 protein and activity levels in frontal neocortical tissue samples from post-mortem AD dementia brains when compared to those with MCI and NCI. Significant correlation between increase in synaptosomal calpain-2 activity and reduced performance of the human subjects in memory and other cognitive tests, indicating that dysregulation of calpain-2 in a synapse-specific manner might potentially contribute to synaptic dysfunction and consequently memory and cognitive deficits that are central to AD pathogenesis. Interestingly, while synaptosomal calpain-2 activity in post-mortem human brains was positively correlated with β-amyloid load, no correlation was observed between synaptosomal calpain-2 activity and hyperphosphorylated tau tangles. Recent studies have shown a positive correlation between levels of truncated calpain-1 and tau phosphorylation, tangle score and Braak stage in human brains^45,46^. It seems likely that synpatosomal calpain-2 hyperactivation which is observed as a consequence of elevated β-amyloid (Fig. 6) occurs early in AD pathogenesis^43^ and calpain-1 hyperactivation which occurs later in the disease progression is the main contributor to tau pathology in AD via the GSK 3β-PP2A pathway^47^ and dual specificity tyrosine-phosphorylation-regulated kinase 1A (Dyrk 1A^48^) pathways.

## Conclusion

In conclusion, we show for the first time that calpain-2 activity is selectively increased at the synapse during prodromal stage in AD mice. Further, we also show elevated synaptosomal calpain-2 protein and activity levels in frontal neocortical tissue samples from post mortem AD dementia brains and its significant correlation with reduced performance in memory and other cognitive tests indicating that activation of calpain-2 might potentially contribute to synaptic dysfunction in AD pathogenesis.

## Electronic supplementary material

Supplementary Data

## Data Availability

The data sets generated during and/or analyzed during the current study are available from the corresponding author on reasonable request.
