# Supplementary material for: Isoform-specific hyperactivation of calpain-2 occurs presymptomatically at the synapse in Alzheimer’s disease mice and correlates with memory deficits in human subjects
Source: Sci Rep. 2018 Sep 3;8:13119. doi: 10.1038/s41598-018-31073-6 (PMC6120938; doi:10.1038/s41598-018-31073-6)
Supplement: Supplementary file 1 — Supplementary Data [file 41598_2018_31073_MOESM1_ESM.pdf]

# Isoform-specific hyperactivation of calpain-2 occurs presymptomatically at the synapse in Alzheimer’s disease mice and correlates with memory deficits in human subjects

Faraz Ahmad

Debajyoti Das

Reddy Peera Kommaddi

Latha Diwakar

Ruturaj Gowaikar

Khader Valli Rupanagudi

David A. Bennett

Vijayalakshmi Ravindranath

# SUPPLEMENTARY DATA

## Supplementary Table 1

| Case | Age at death (yr.) | Gender | PMI (h) | Braak stage |
|---|---|---|---|---|
| NCI 1 | 77.31 | female | 5.83 | I |
| NCI 2 | 93.64 | female | 12.42 | I |
| NCI 3 | 91.14 | female | 16.33 | 0 |
| NCI 4 | 78.67 | male | 7.00 | I |
| NCI 5 | 87.87 | male | 6.17 | I |
| NCI 6 | 85.83 | male | 7.67 | I |
| NCI 7 | 71.66 | male | 5.43 | 0 |
| NCI 8 | 81.16 | male | 12.43 | I |
| NCI 9 | 96.02 | female | 4.17 | I |
| NCI 10 | 83.37 | female | 1.00 | I |
| NCI 11 | 80.85 | female | 4.83 | I |
| NCI 12 | 75.85 | female | 3.00 | I |
| **MEAN ± SD** | 83.61 ± 7.48 | | 7.19 ± 4.42 | |
| | | | | |
| MCI 1 | 78.73 | female | 13.58 | 0 |
| MCI 2 | 91.28 | female | 6.38 | IV |
| MCI 3 | 93.63 | female | 12.33 | II |
| MCI 4 | 74.96 | male | 16.00 | III |
| MCI 5 | 85.85 | male | 10.63 | IV |
| MCI 6 | 89.78 | male | 20.88 | IV |
| MCI 7 | 85.32 | male | 5.00 | III |
| MCI 8 | 96.32 | male | 5.92 | III |
| MCI 9 | 90.70 | female | 4.33 | III |
| MCI 10 | 99.36 | female | 4.33 | III |
| MCI 11 | 90.67 | female | 4.55 | III |
| MCI 12 | 80.09 | female | 4.78 | III |
| **MEAN ± SD** | 88.06 ± 7.32 | | 9.06 ± 5.55 | |
| | | | | |
| AD 1 | 80.64 | female | 6.00 | V |
| AD 2 | 90.10 | female | 4.97 | V |
| AD 3 | 93.24 | female | 18.17 | IV |
| AD 4 | 92.40 | male | 12.9 | III |
| AD 5 | 90.31 | male | 2.75 | V |
| AD 6 | 85.11 | male | 5.00 | V |
| AD 7 | 92.33 | female | 7.42 | V |
| AD 8 | 92.51 | female | 17.58 | V |
| AD 9 | 94.98 | female | 5.25 | V |
| AD 10 | 89.66 | female | 11.25 | V |
| AD 11 | 88.18 | female | 5.08 | V |
| AD 12 | 94.75 | female | 3.12 | V |
| **MEAN ± SD** | 90.35 ± 4.14 | | 8.29 ± 5.38 | |

**Supplementary Table 1. Details of patients with no cognitive impairment (NCI), mild cognitive impairment (MCI) and Alzheimer's disease dementia (AD).**

PMI: post-mortem interval

**Supplementary Figure 1**

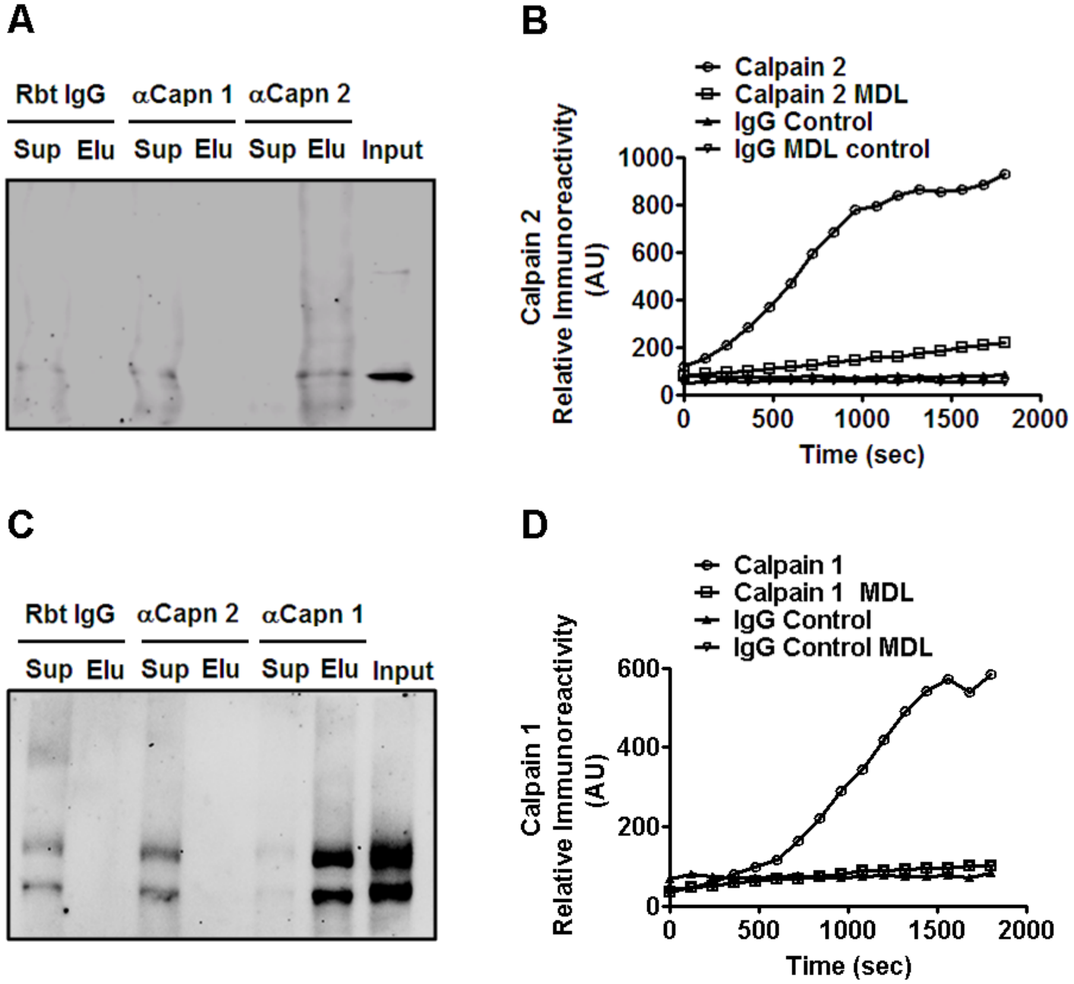


**Supplementary Fig. 1. Immunoprecipitation was employed to assay isoform specific calpain-2 and calpain-1 activities in synaptosomes.**

**(A)** Use of calpain-2 specific antibody allowed immunoprecipitation of calpain-2 (as seen as calpain-2 immunoreactive band in the eluent fraction) from synaptosomal samples prepared from mouse brain cortex. Normal rabbit IgG used as a negative control did not pull down calpain-2. Calpain-1 antibody used for immunoprecipitation was also unable to pull down calpain-2. 'Elu' represents eluent or immunoprecipitated fraction and 'Sup' represents supernatant or unbound fraction. 'Input' was used a

marker for calpain-2. **(B)** Use of fluoregenic Suc-LLVY-AMC peptide substrate confirmed synaptosomal calpain-2 activation in samples immunoprecipitated with anti-calpain-2 antibody but not with normal rabbit IgG. Calpain inhibitor MDL 28170 blocked calpain-2 activation. **(C)** Anti-calpain-1 but not anti-calpain 2 antibody could immunoprecipitate calpain-1 from synaptosomal samples prepared from mouse brain cortex. Normal rabbit IgG was used as a negative control and did not pull down calpain-1. ‘Elu’ represents eluent or immunoprecipitated fraction and ‘Sup’ represents supernatant or unbound fraction. ‘Input’ was used a marker for calpain-1. **(D)** Fluoregenic Suc-LLVY-AMC peptide substrate was used to confirm activation of synaptosomal calpain-1 in samples immunoprecipitated with anti-calpain-1 antibody but not with normal rabbit IgG. Calpain inhibitor MDL 28170 blocked calpain-1 activation.

**Supplementary Figure 2**

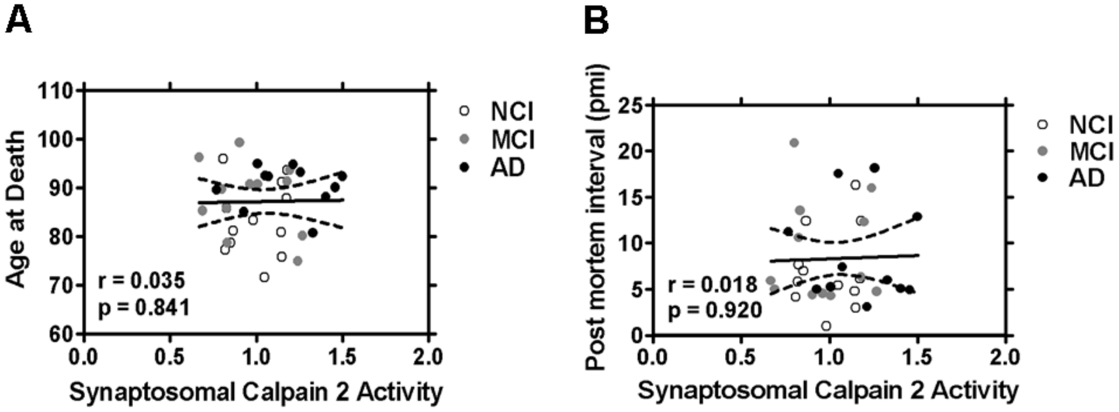


**Supplementary Fig. 2. Activity levels of synaptosomal calpain-2 of frontal neocortical tissue samples of post-mortem brains from human subjects are not affected by post mortem indices or age at death.**

No correlation was observed between synaptosomal calpain-2 activity and age of death **(A)** and post mortem index (pmi; **(B)**) of the subjects.

**Supplementary Figure 3. Representative full blots for data in Figure 3A.**

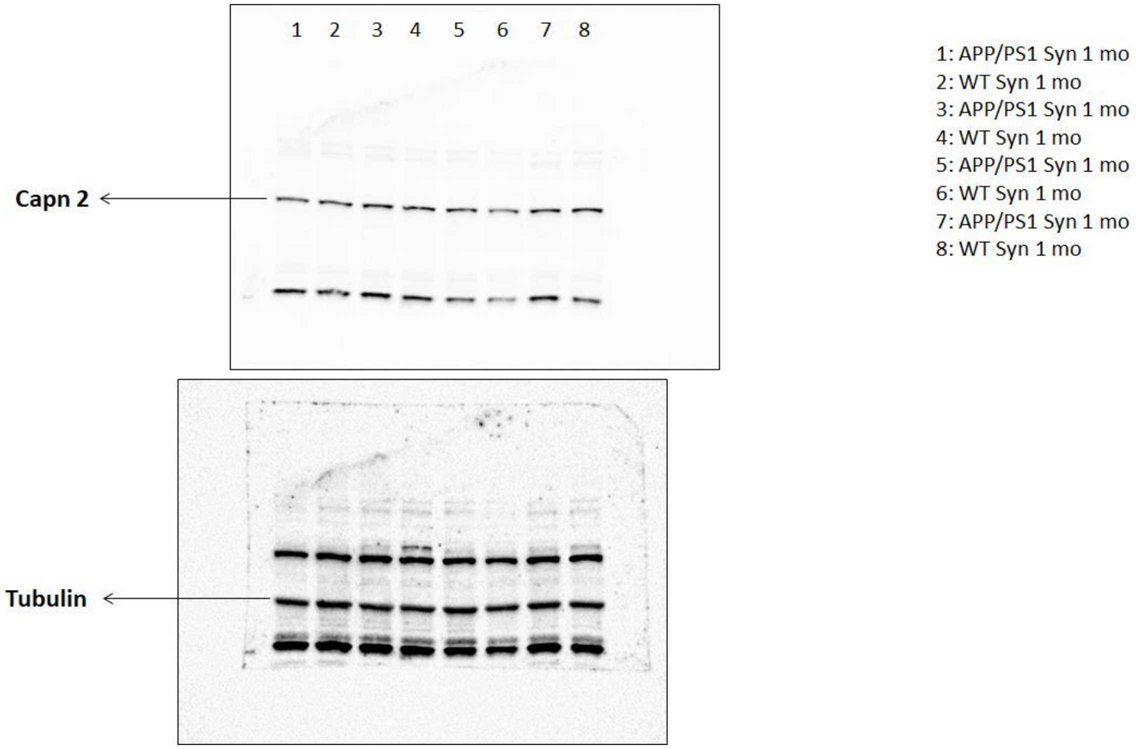

Capn 2

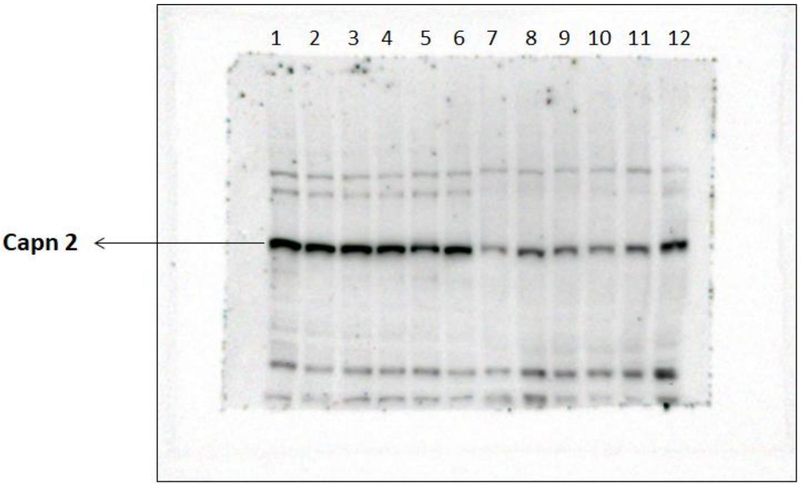


Tubulin

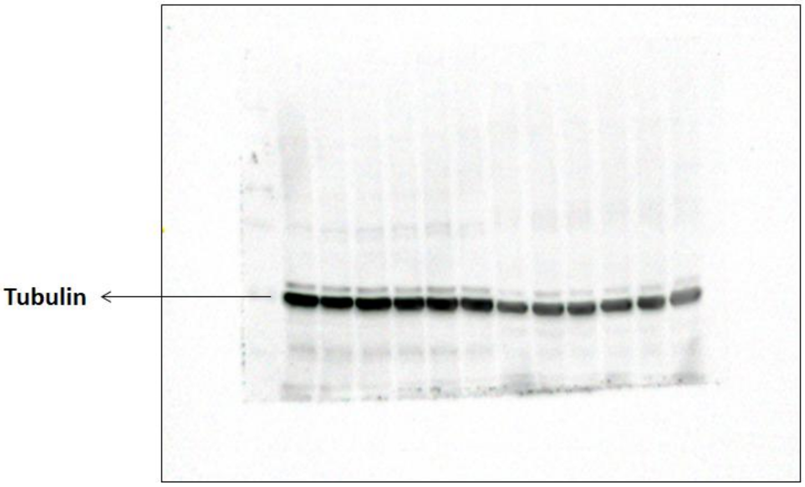

1: WT PNS 3 mo
2: APP/PS1 PNS 3 mo
3: WT PNS 3 mo
4: APP/PS1 PNS 3 mo
5: APP/PS1 PNS 3 mo
6: WT PNS 3 mo
7: WT Syn 1 mo
8: APP/PS1 Syn 1 mo
9: WT Syn 1 mo
10: APP/PS1 Syn 1 mo
11: WT Syn 1 mo
12: APP/PS1 Syn 1 mo

**Supplementary Figure 4. Representative full blots for data in Figure 3B.**

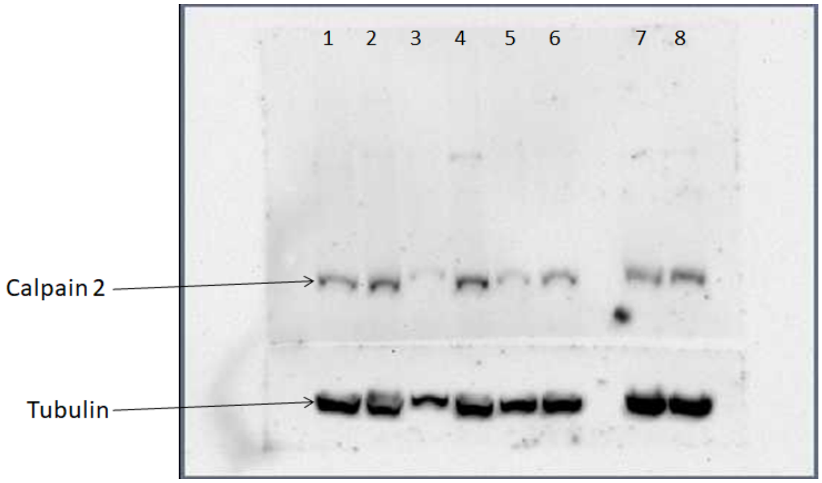


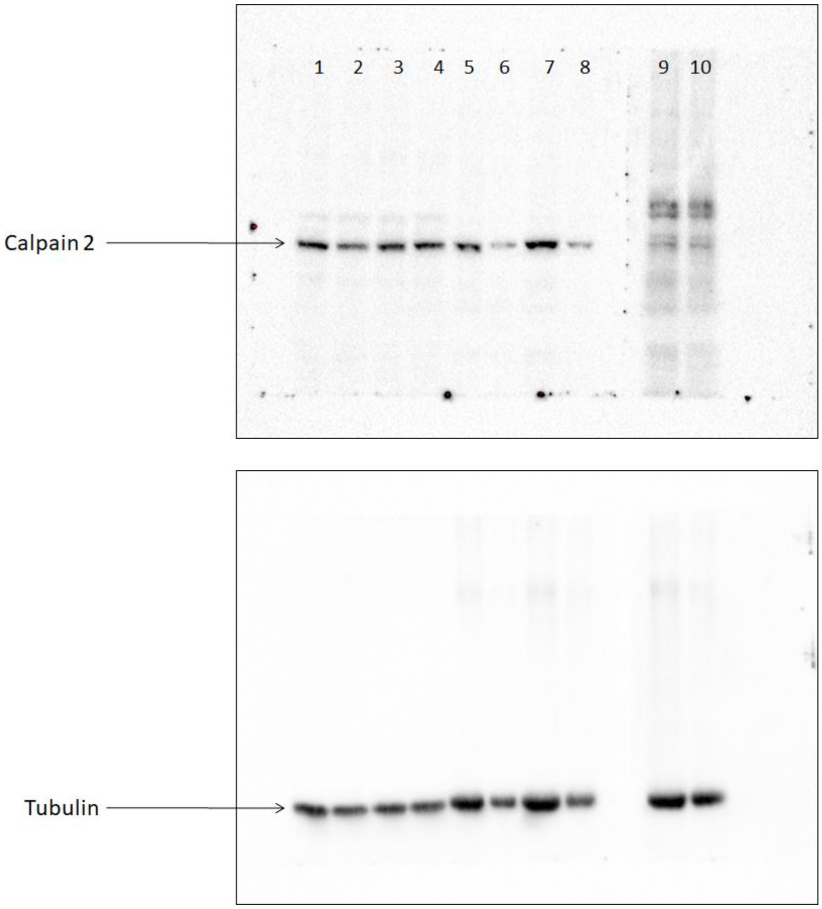

**Supplementary Figure 5. Representative full blots for data in Figure 3C.**

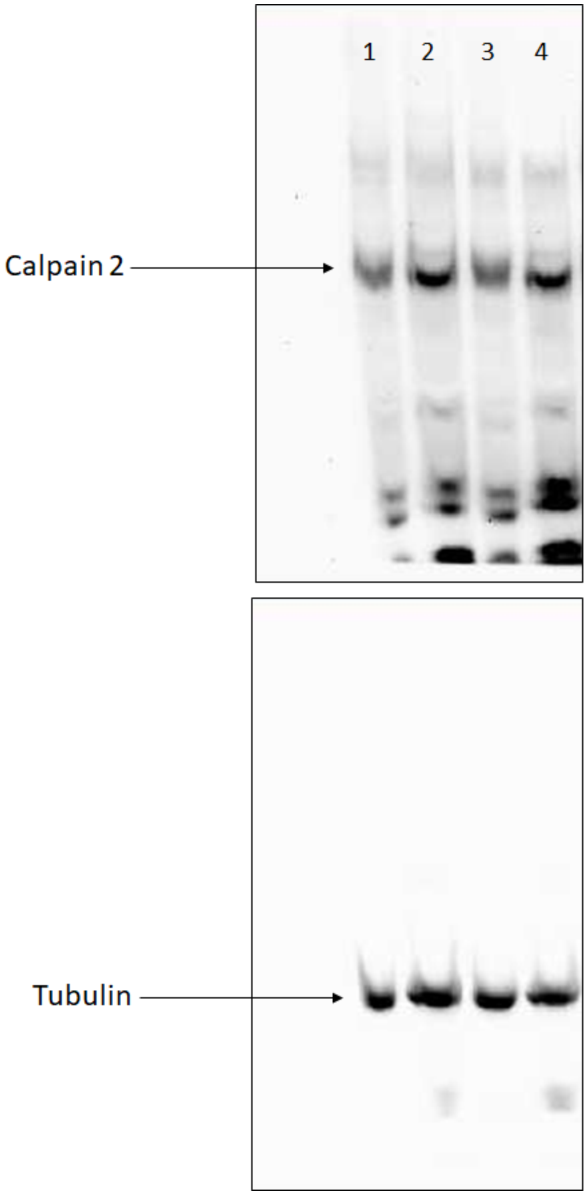


1: WT Syn 10 mo
2 APP/PS1 Syn 10 mo
3: WT Syn 10 mo
4: APP/PS1 Syn 10 mo
5: WT Syn 10 mo
6: APP/PS1 Syn 10 mo

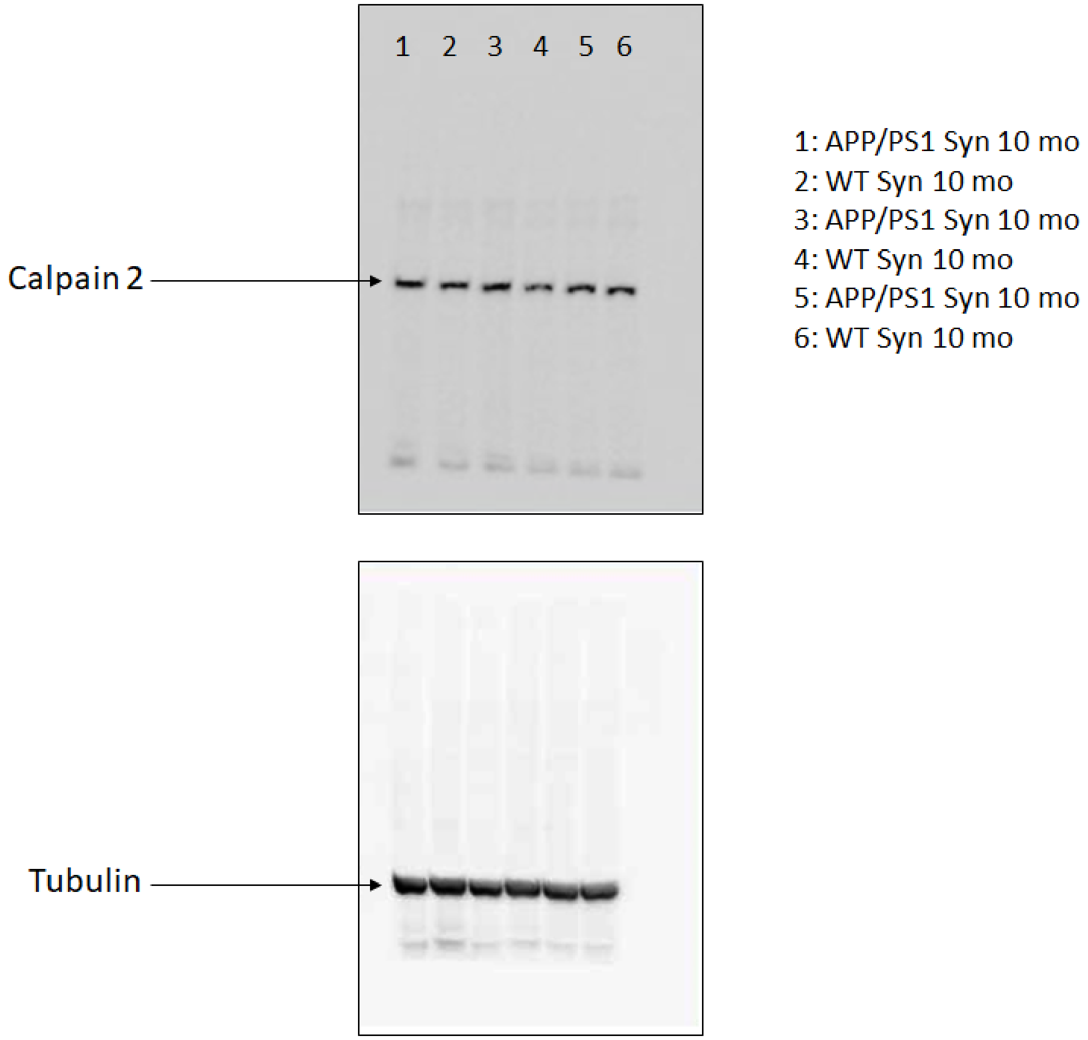

1 2 3 4 5 6
Calpain 2
Tubulin
1: APP/PS1 Syn 10 mo
2: WT Syn 10 mo
3: APP/PS1 Syn 10 mo
4: WT Syn 10 mo
5: APP/PS1 Syn 10 mo
6: WT Syn 10 mo

**Supplementary Figure 6. Representative full blots for data in Figure 4A.**

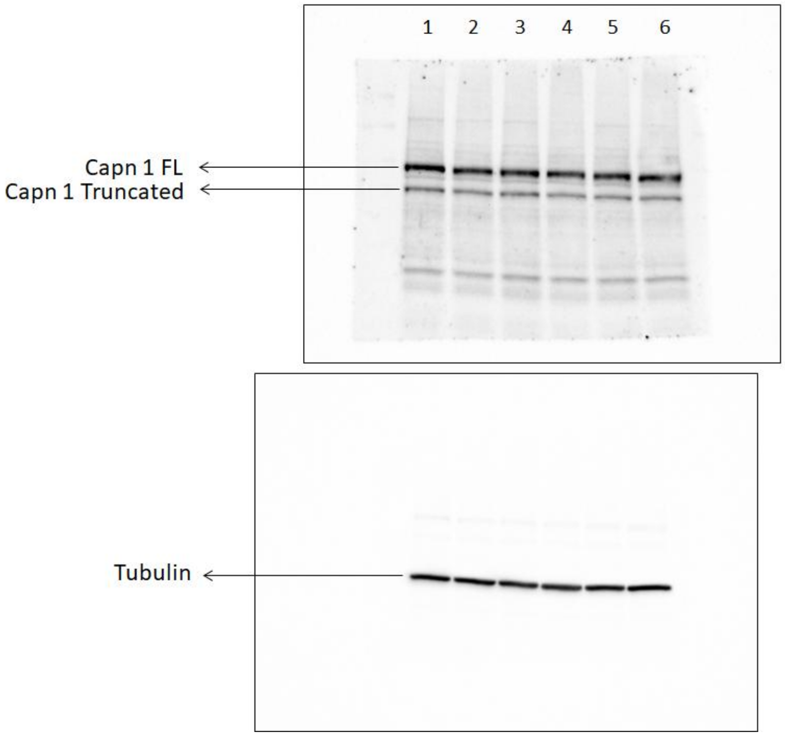


1: WT Syn 1 mo
2: APP/PS1 Syn 1 mo
3: WT Syn 1 mo
4: APP/PS1 Syn 1 mo
5: WT Syn 1 mo
6: APP/PS1 Syn 1 mo

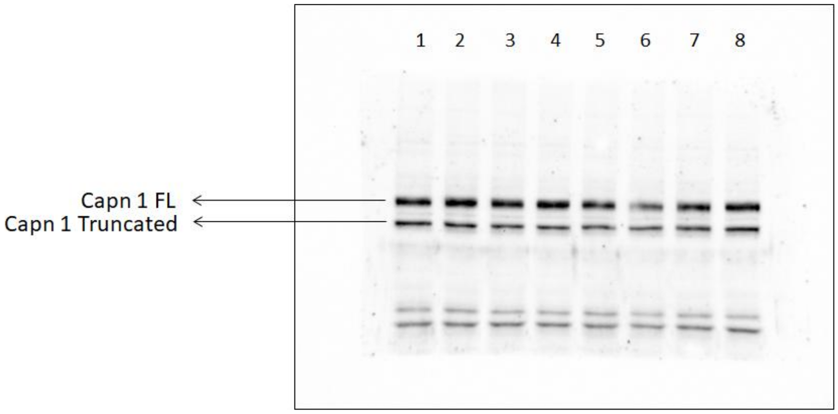


1: APP/PS1 Syn 1 mo
2: WT Syn 1 mo
3: APP/PS1 Syn 1 mo
4: WT Syn 1 mo
5: APP/PS1 Syn 1 mo
6: WT Syn 1 mo
7: APP/PS1 Syn 1 mo
8: WT Syn 1 mo

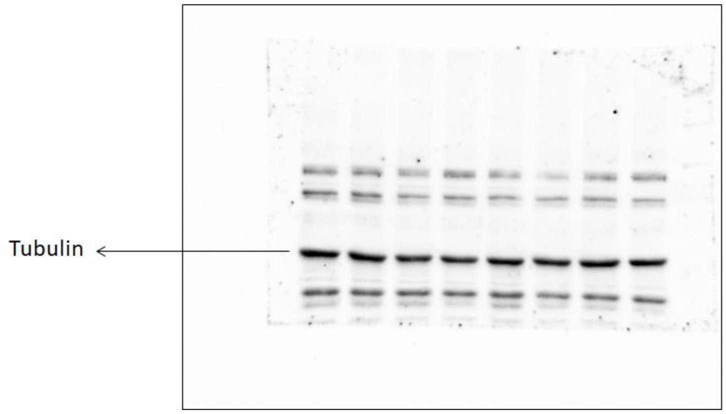

**Supplementary Figure 7. Representative full blots for data in Figure 4B.**

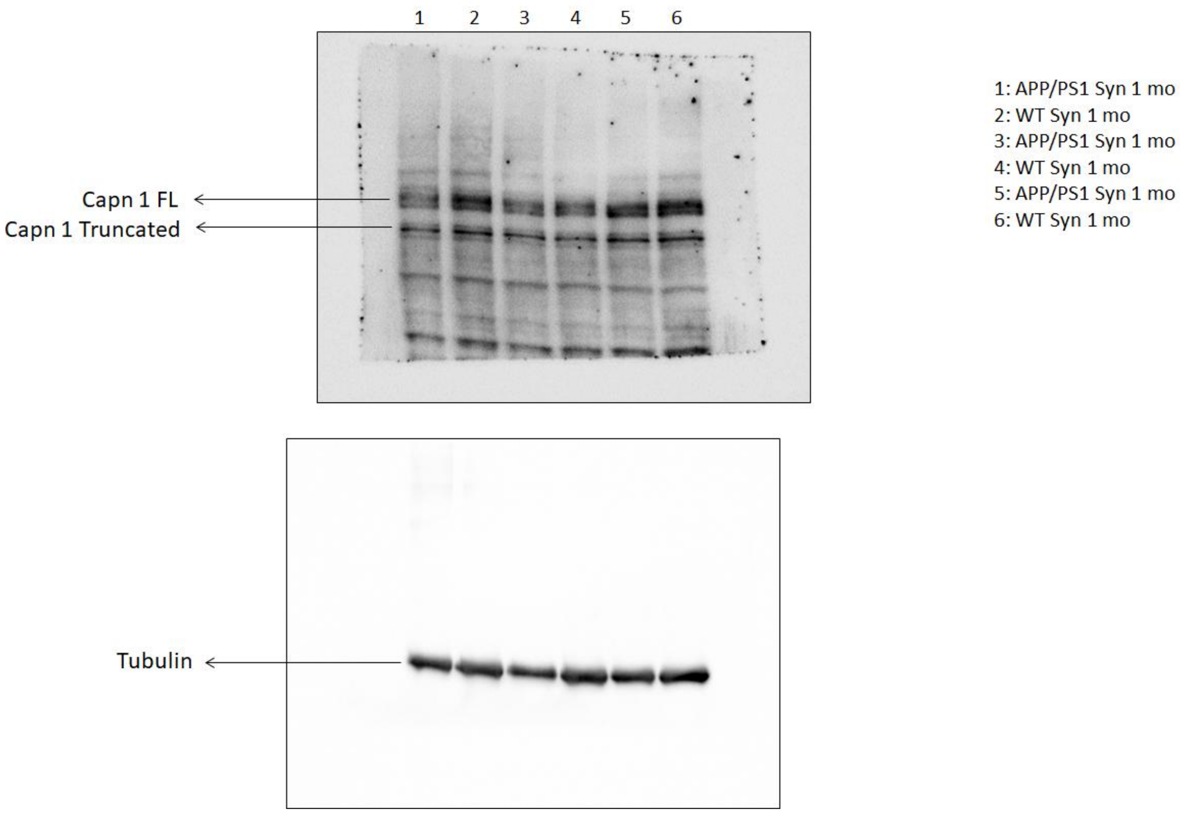

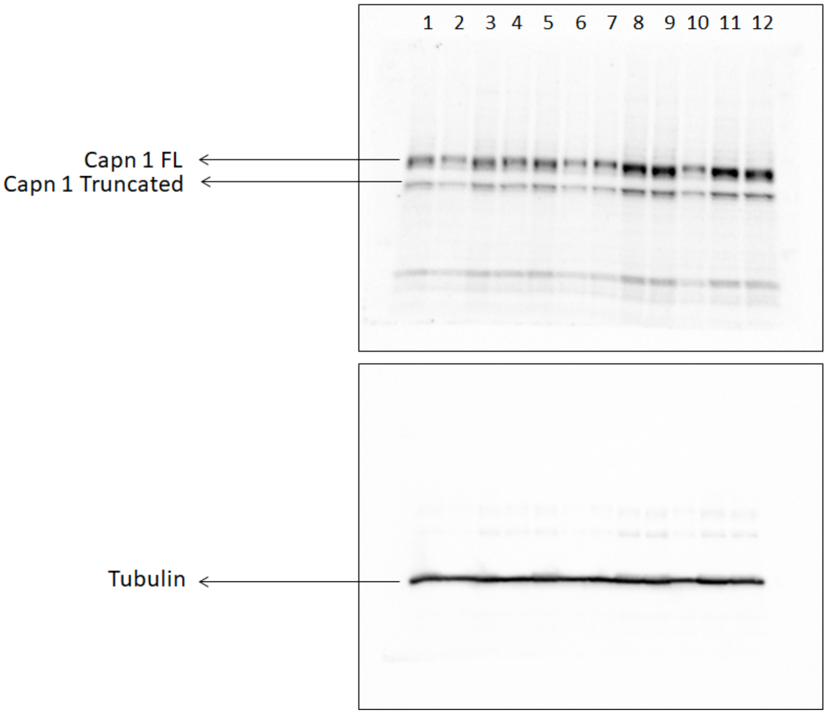


1: APP/PS1 Syn 3 mo
2: WT Syn 3 mo
3: APP/PS1 Syn 3 mo
4: WT Syn 3 mo
5: APP/PS1 Syn 3 mo
6: WT Syn 3 mo
7: APP/PS1 Syn 3 mo
8: WT Syn 3 mo
9: APP/PS1 Syn 3 mo
10: WT Syn 3 mo
11: APP/PS1 Syn 3 mo
12: WT Syn 3 mo

**Supplementary Figure 8. Representative full blots for data in Figure 4C.**

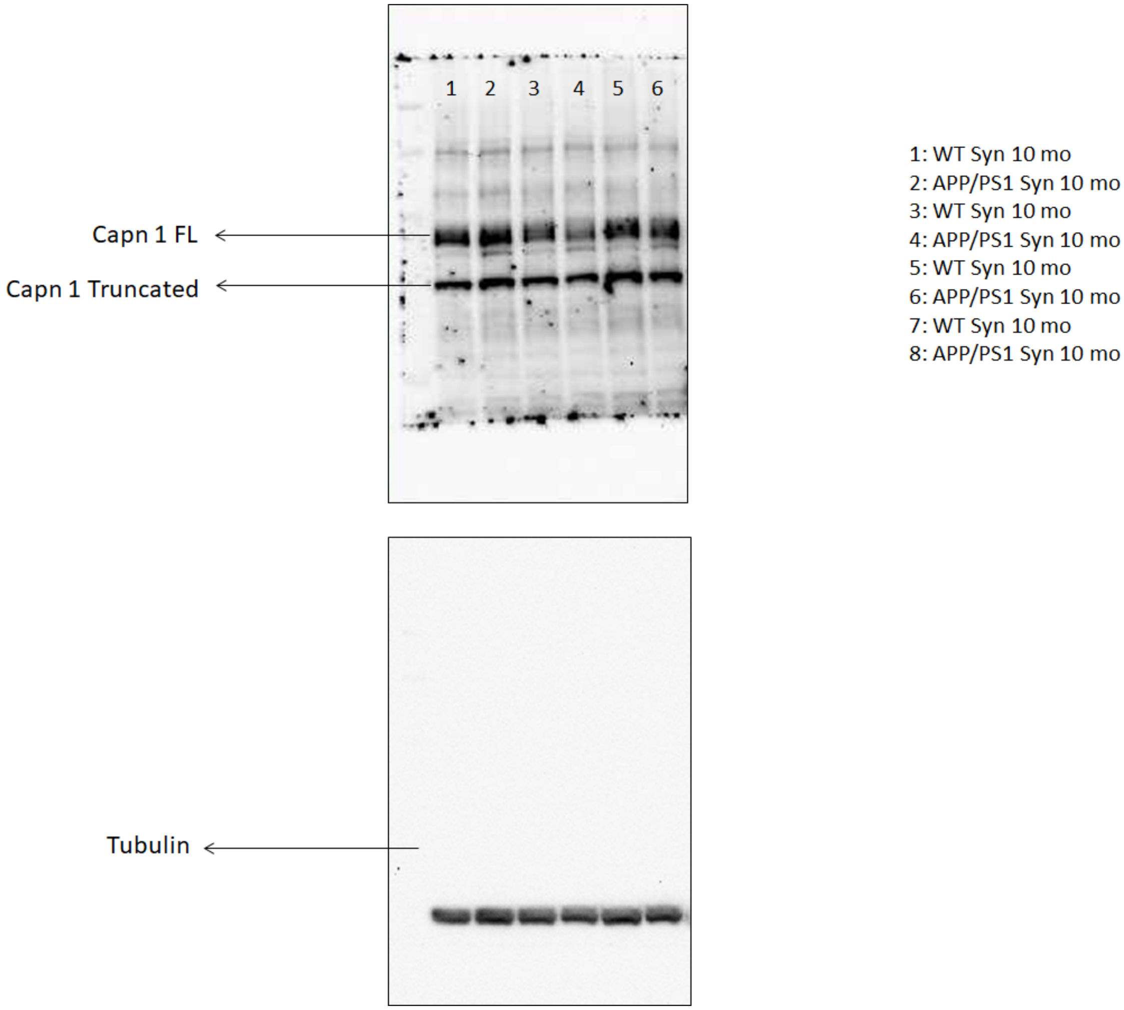

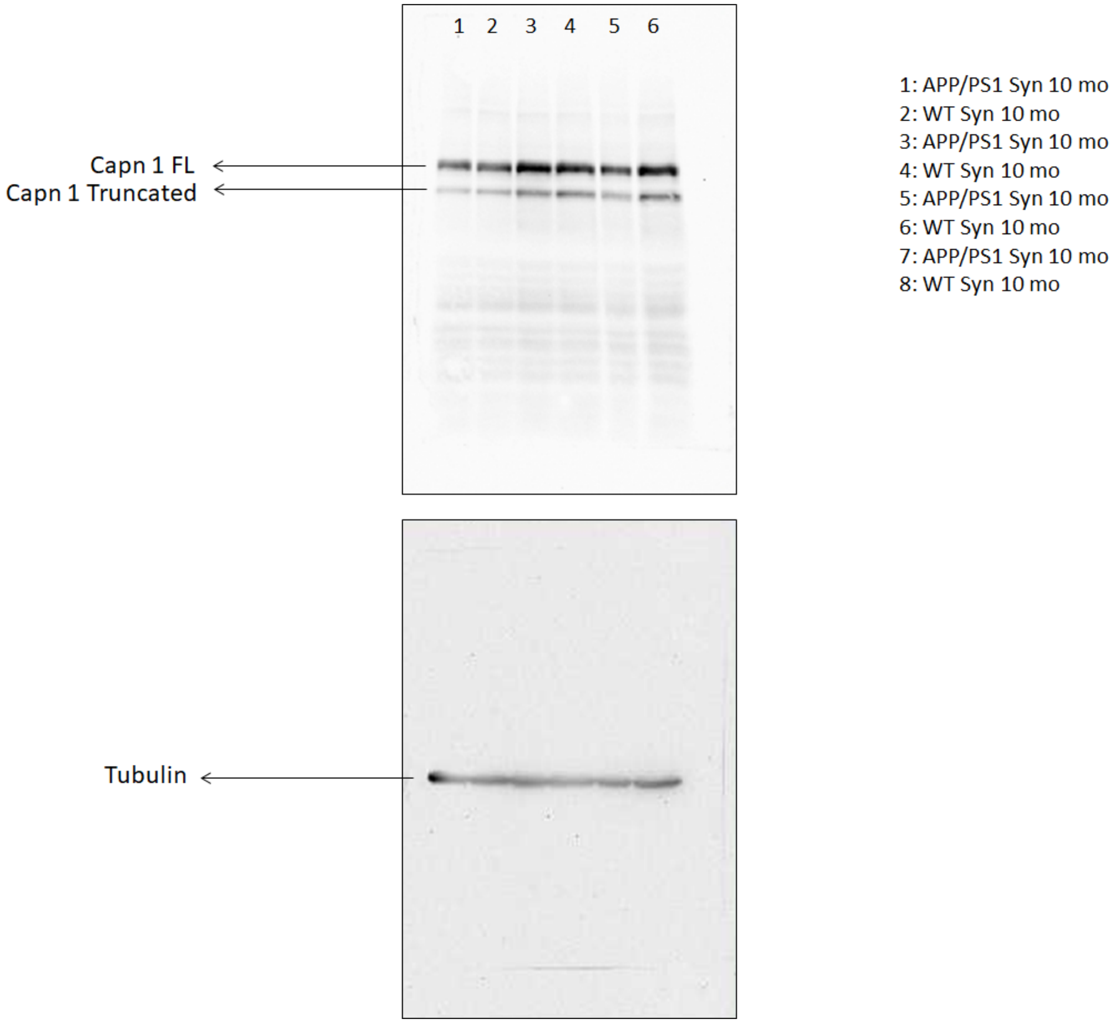
1
2
3
4
5
6
Capn 1 FL
Capn 1 Truncated
Tubulin
1: APP/PS1 Syn 10 mo
2: WT Syn 10 mo
3: APP/PS1 Syn 10 mo
4: WT Syn 10 mo
5: APP/PS1 Syn 10 mo
6: WT Syn 10 mo
7: APP/PS1 Syn 10 mo
8: WT Syn 10 mo

**Supplementary Figure 9. Representative full blots for data in Figure 5A.**

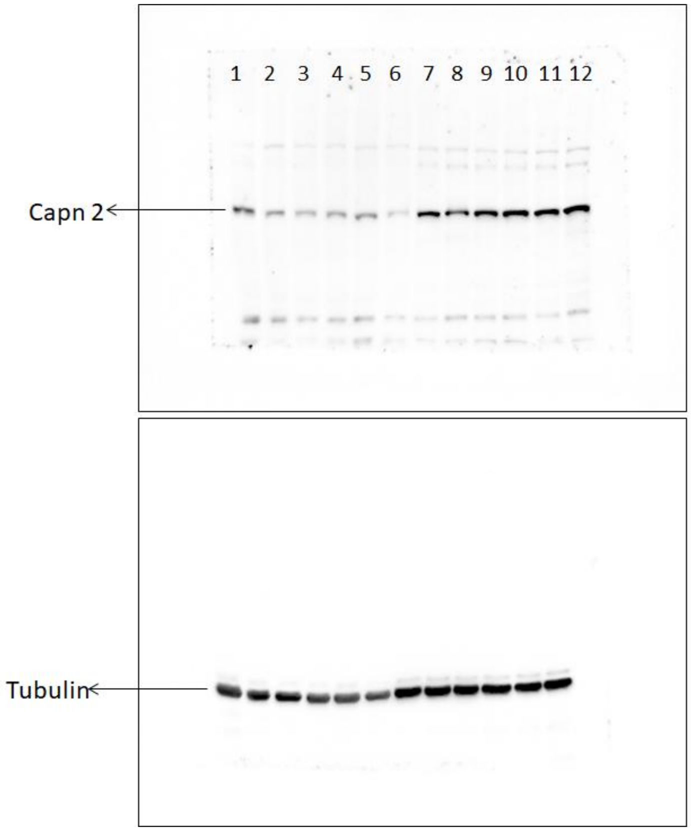


1: APP/PS1 Syn 1 mo
2: WT Syn 1 mo
3: APP/PS1 Syn 1 mo
4: WT Syn 1 mo
5: APP/PS1 Syn 1 mo
6: WT Syn 1 mo

7: WT PNS 1 mo
8: APP/PS1 PNS 1 mo
9: APP/PS1 PNS 1 mo
10: WT PNS 1 mo
11: APP/PS1 PNS 1 mo
12: WT PNS 1 mo

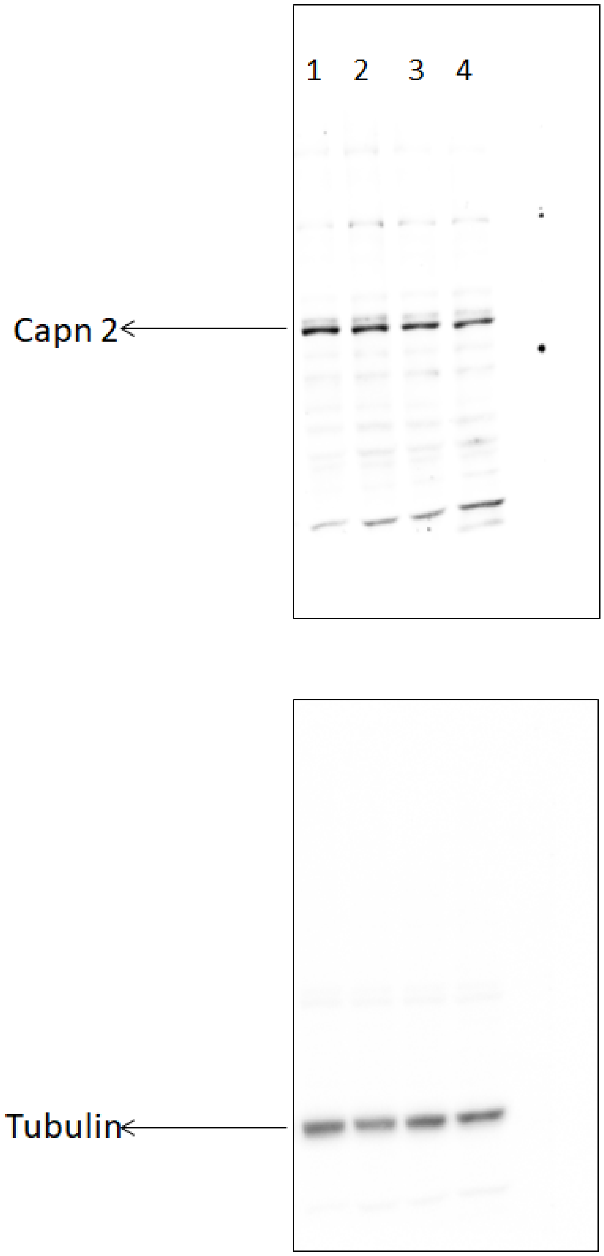


1: APP/PS1 PNS 1 mo
2: WT PNS 1 mo
3: APP/PS1 PNS 1 mo
4: WT PNS 1 mo

**Supplementary Figure 10. Representative full blots for data in Figure 5B.**

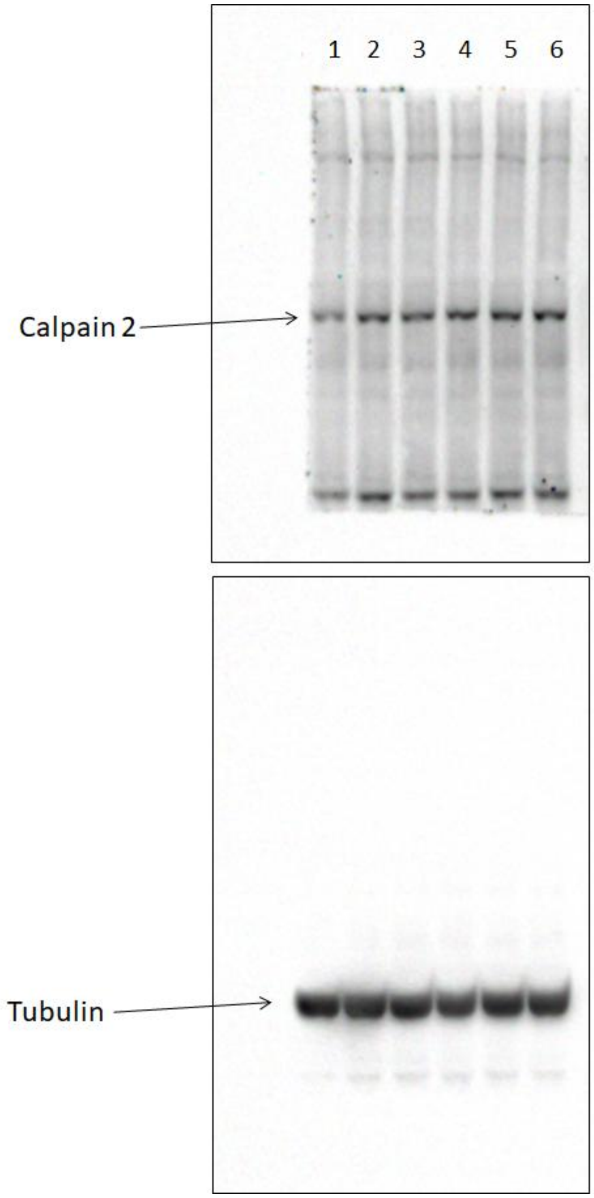


1: APP/PS1 PNS 3 mo
2: WT PNS 3 mo
3: APP/PS1 PNS 3 mo
4: WT PNS 3 mo
5: APP/PS1 PNS 3 mo
6: WT PNS 3 mo

7: APP/PS1 Syn 1 mo
8: WT Syn 1 mo
9: APP/PS1 Syn 1 mo
10: WT Syn 1 mo
11: APP/PS1 Syn 1 mo
12: WT Syn 1 mo

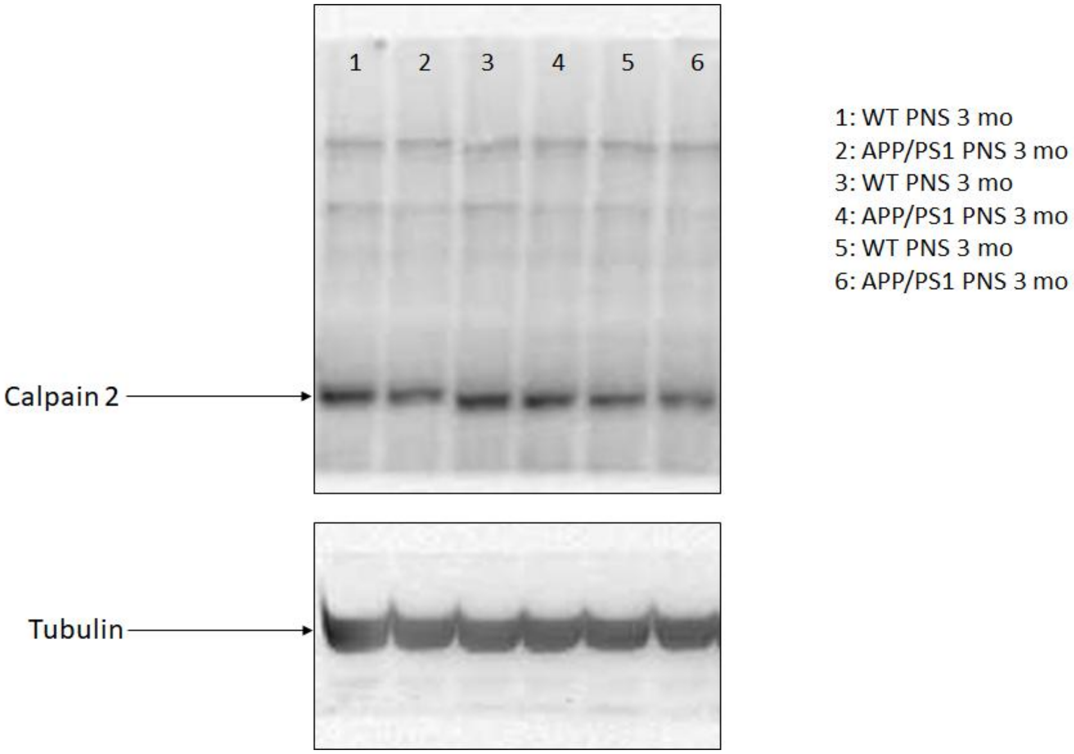

1
2
3
4
5
6
Calpain 2
Tubulin
1: WT PNS 3 mo
2: APP/PS1 PNS 3 mo
3: WT PNS 3 mo
4: APP/PS1 PNS 3 mo
5: WT PNS 3 mo
6: APP/PS1 PNS 3 mo

**Supplementary Figure 11. Representative full blots for data in Figure 5C.**

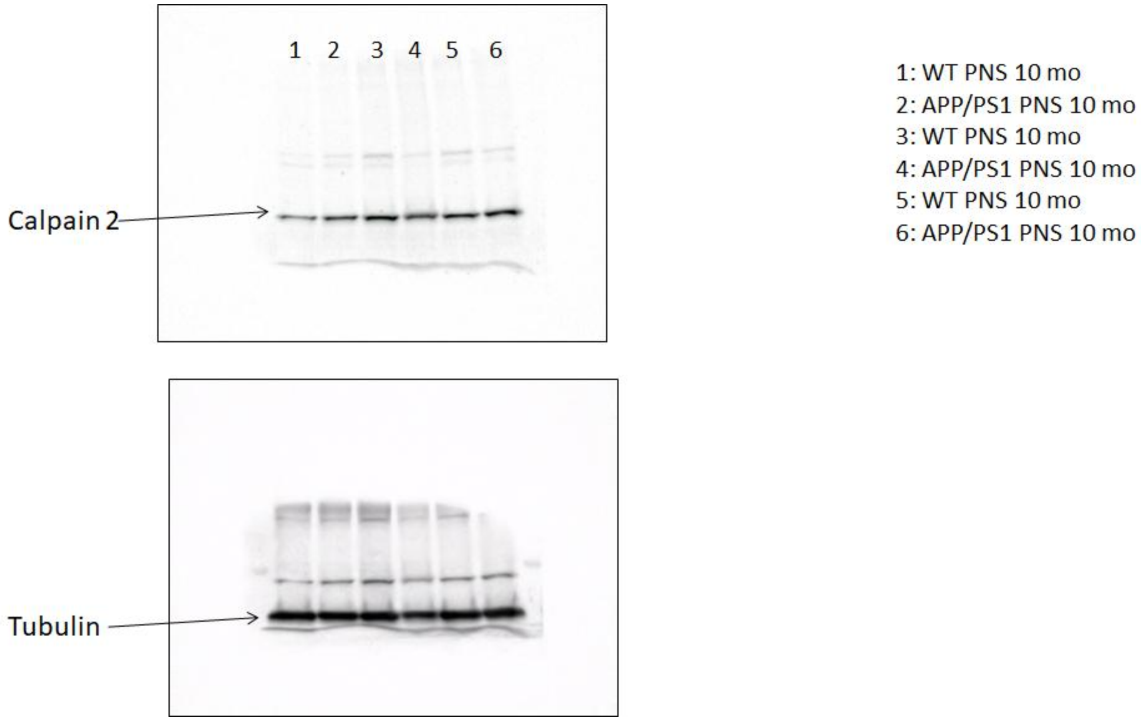

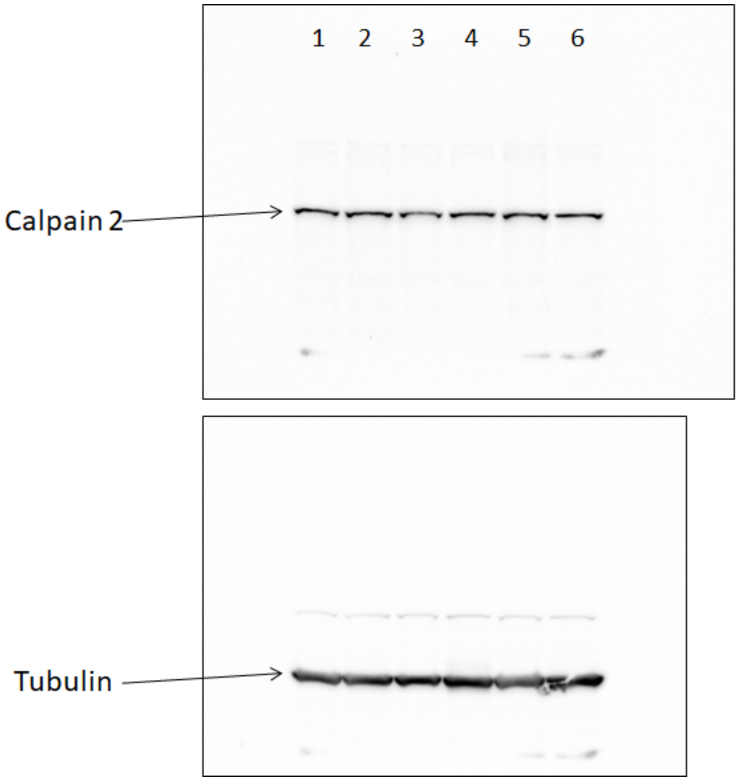


1: WT PNS 10 mo
2: APP/PS1 PNS 10 mo
3: WT PNS 10 mo
4: APP/PS1 PNS 10 mo
5: WT PNS 10 mo
6: WT PNS 10 mo

**Supplementary Figure 12. Representative full blots for data in Figure 7A.**

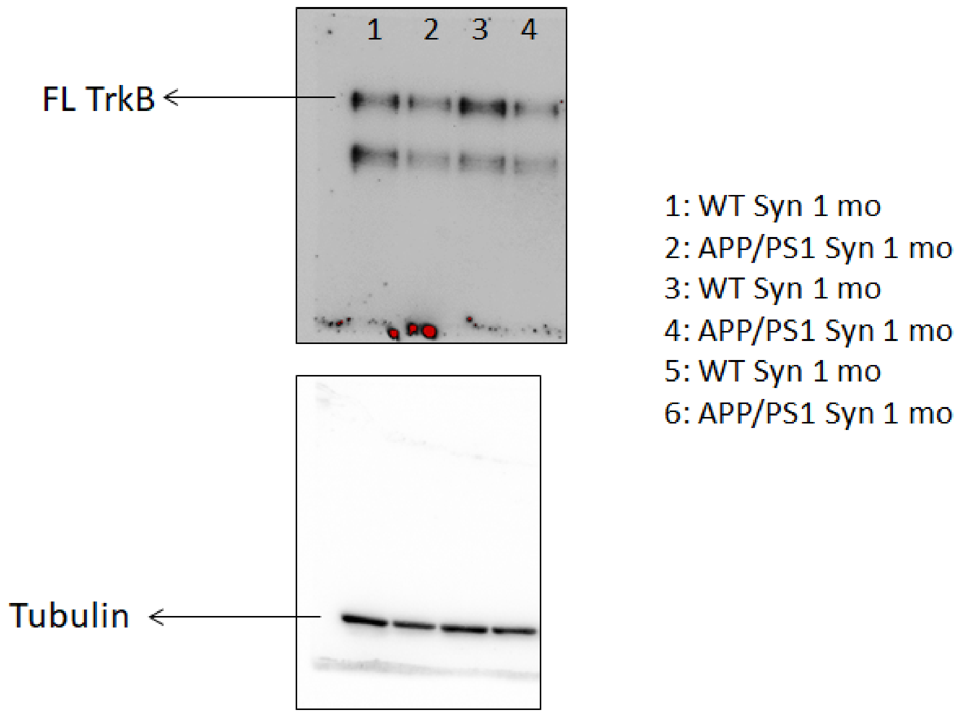

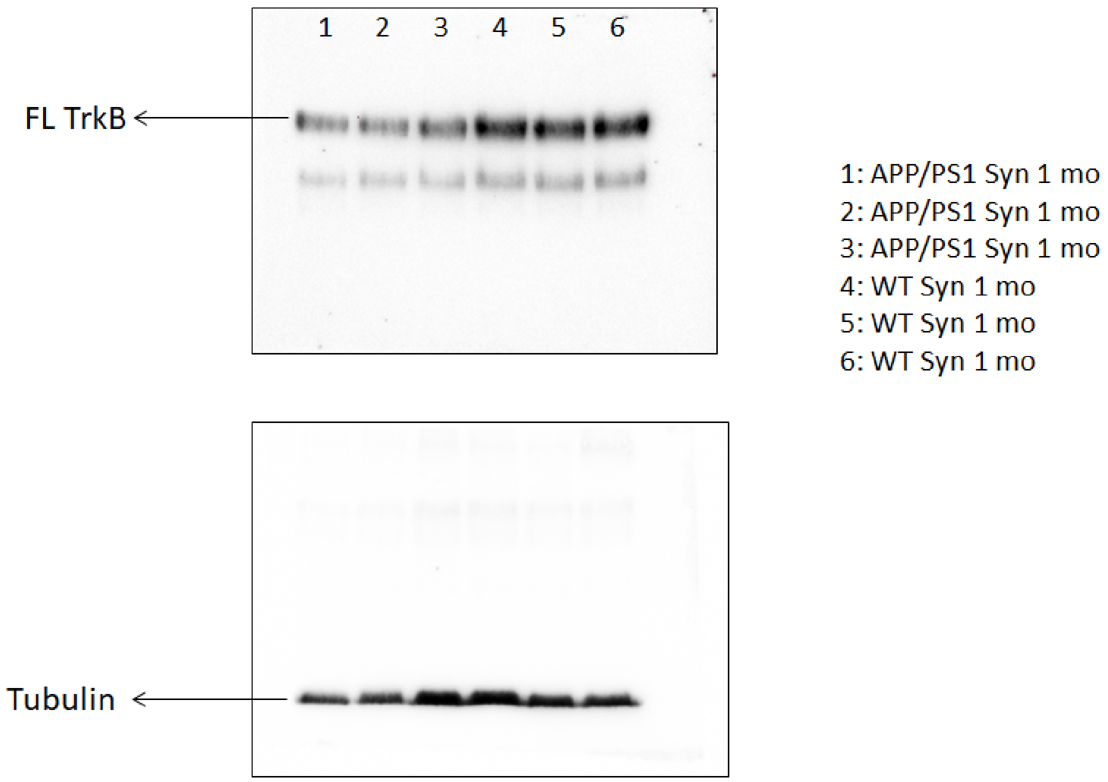
1
2
3
4
5
6
FL TrkB
Tubulin
1: APP/PS1 Syn 1 mo
2: APP/PS1 Syn 1 mo
3: APP/PS1 Syn 1 mo
4: WT Syn 1 mo
5: WT Syn 1 mo
6: WT Syn 1 mo

**Supplementary Figure 13. Representative full blots for data in Figure 7B.**

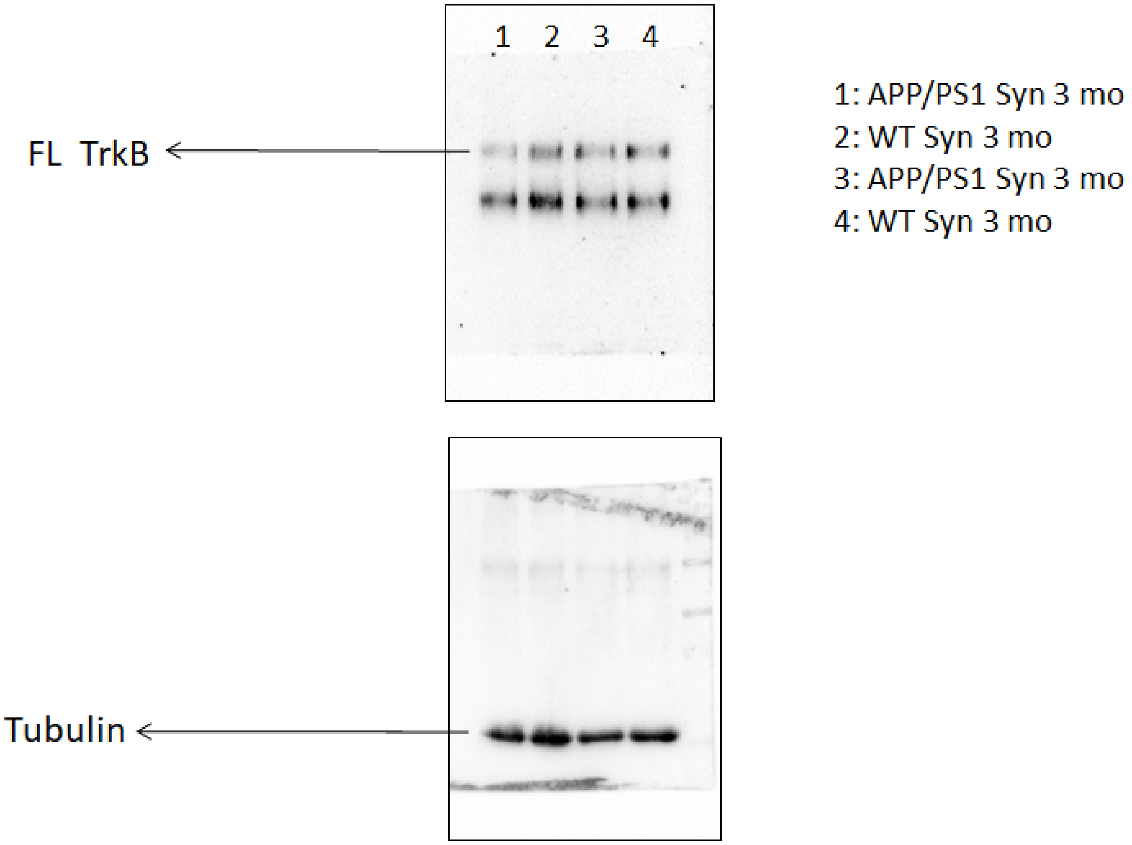

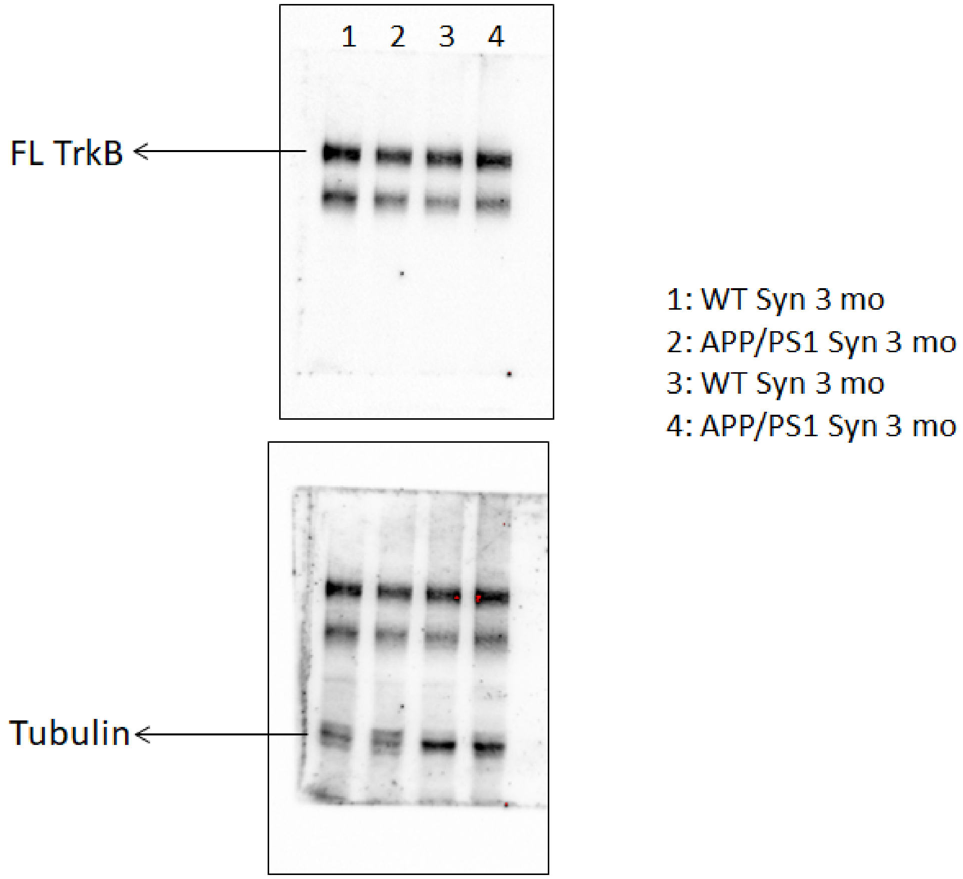
1 2 3 4
FL TrkB
Tubulin
1: WT Syn 3 mo
2: APP/PS1 Syn 3 mo
3: WT Syn 3 mo
4: APP/PS1 Syn 3 mo

**Supplementary Figure 14. Representative full blots for data in Figure 7C.**

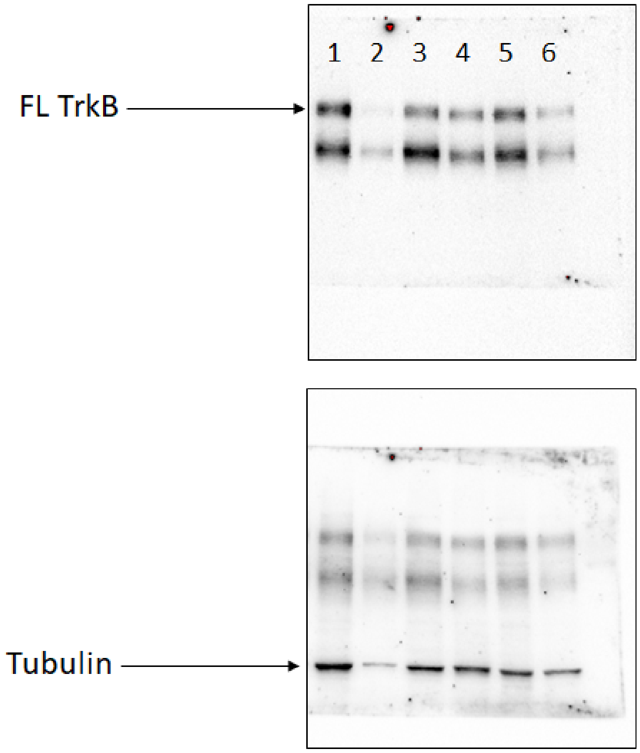


1: WT Syn 10 mo
2: APP/PS1 Syn 10 mo
3: WT Syn 10 mo
4: APP/PS1 Syn 10 mo
5: WT Syn 10 mo
6: APP/PS1 Syn 10 mo
7: WT Syn 10 mo
8: APP/PS1 Syn 10 mo

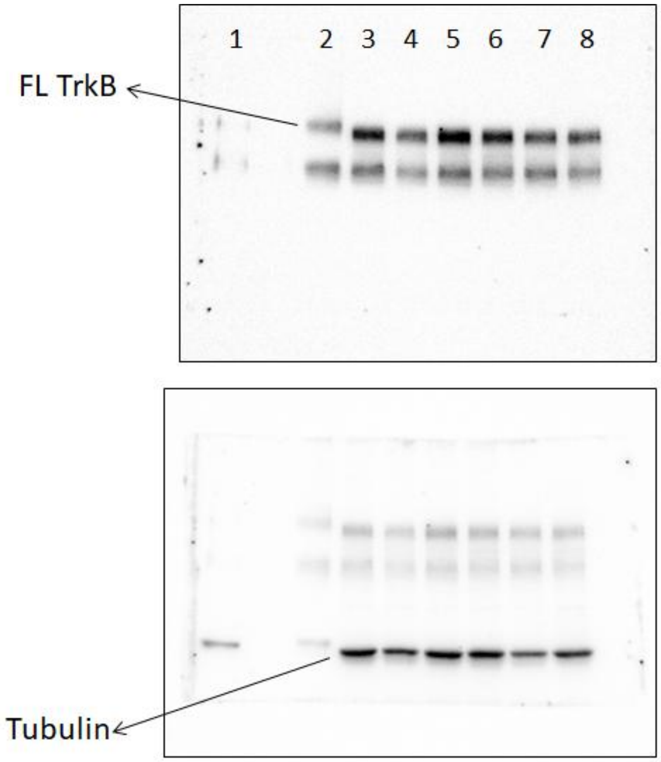


1: Human Syn test
2: Human PNS test

3: WT Syn 10 mo
4: APP/PS1 Syn 10 mo
5: WT Syn 10 mo
6: WT Syn 10 mo
7: APP/PS1 Syn 10 mo
8: APP/PS1 Syn 10 mo

**Supplementary Figure 15. Representative full blots for data in Figure 9A.**

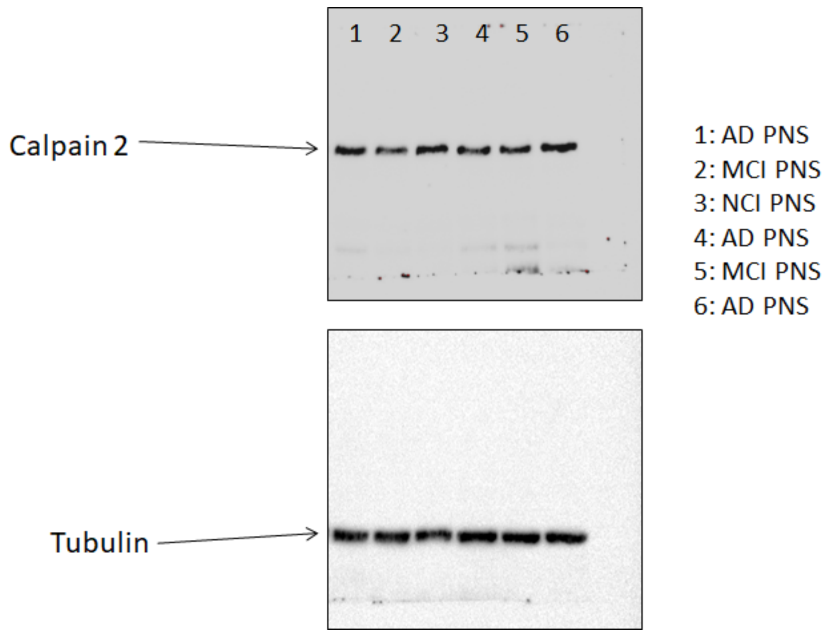

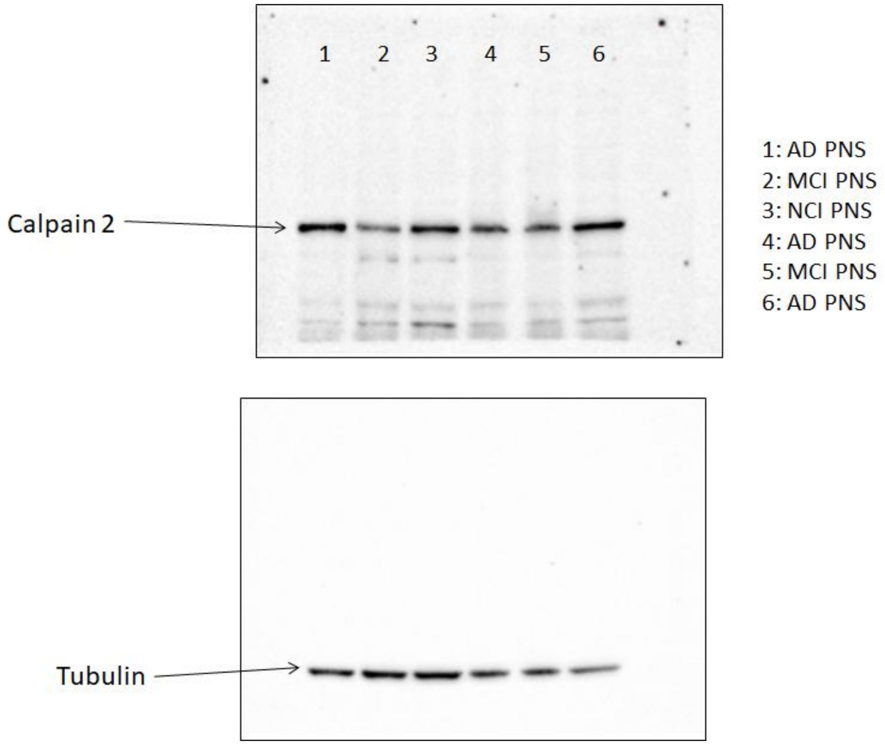
1 2 3 4 5 6
Calpain 2
1: AD PNS
2: MCI PNS
3: NCI PNS
4: AD PNS
5: MCI PNS
6: AD PNS
Tubulin

**Supplementary Figure 16. Representative full blots for data in Figure 9B.**

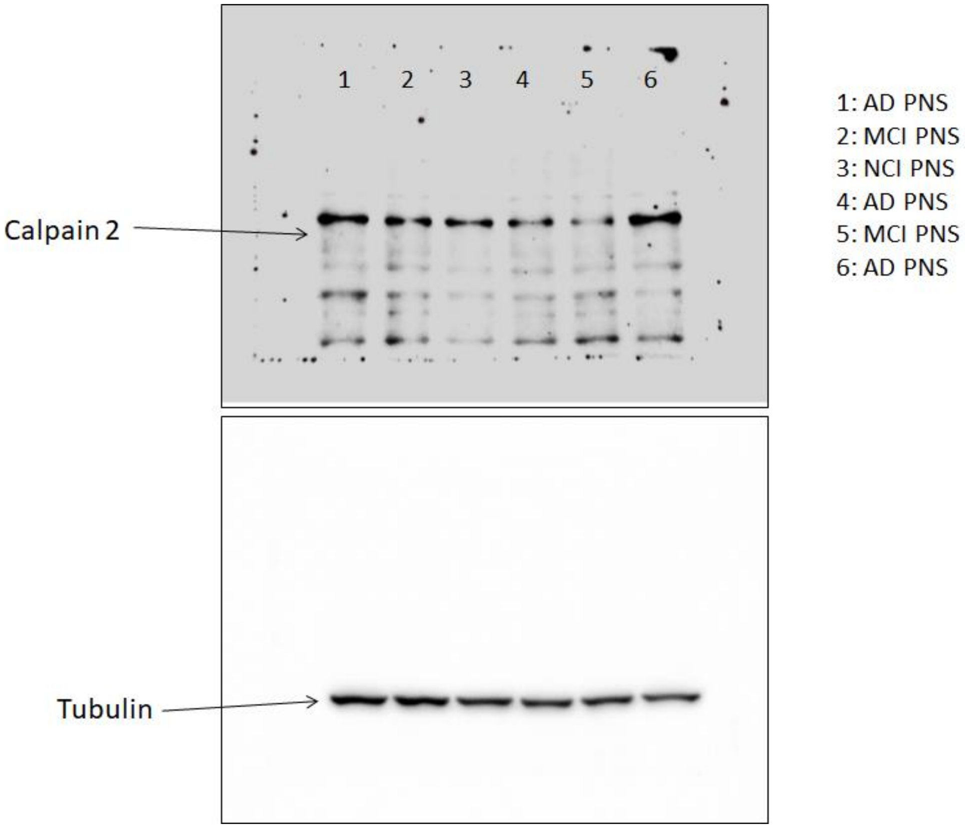

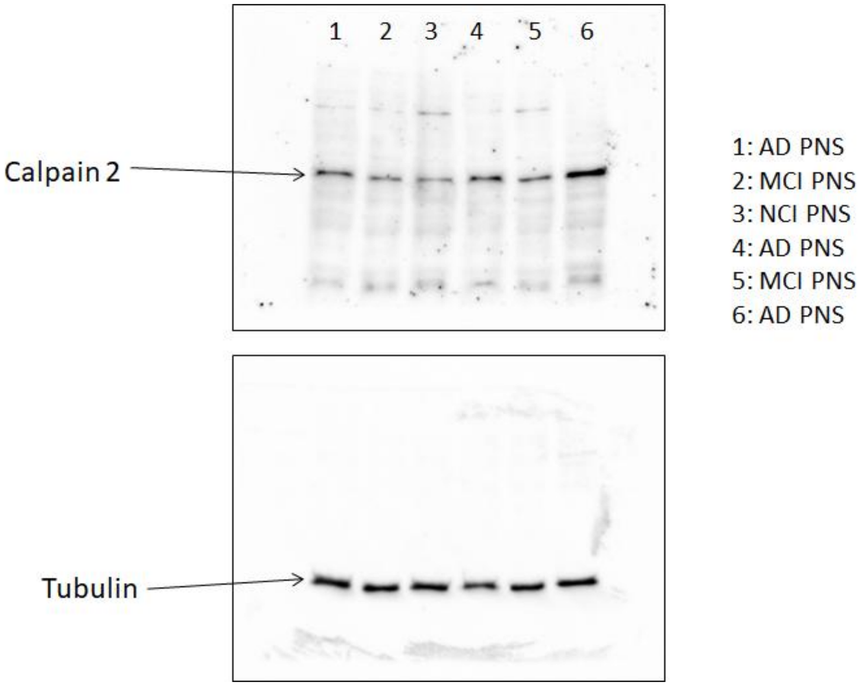

1 2 3 4 5 6
Calpain 2
Tubulin
1: AD PNS
2: MCI PNS
3: NCI PNS
4: AD PNS
5: MCI PNS
6: AD PNS
